# MNP–cellulose–OSO_3_H as an efficient and biodegradable heterogeneous catalyst for green synthesis of trisubstituted imidazoles†

**DOI:** 10.1039/d2ra01348g

**Published:** 2022-04-19

**Authors:** Shaghayegh Saeedi, Abbas Rahmati

**Affiliations:** Department of Chemistry, University of Isfahan P. O. Box 81746-73441 Isfahan Iran a.rahmati@sci.ui.ac.ir +98 31 37934943

## Abstract

Cellulose is an eco-friendly, efficient, and suitable substrate for use as a coating material and support in the preparation of catalysts. Herein, MNP–cellulose–OSO_3_H was prepared as an efficient heterogeneous catalyst composed of Fe_3_O_4_ nanoparticles covered with cellulose–OSO_3_H and used for the synthesis of trisubstituted imidazoles. The catalyst was characterized by FT-IR, CHNS, ICP, PXRD, EDAX, elemental mapping, SEM, TEM, zeta potential, TGA, and VSM techniques. The catalytic activity was evaluated in the one-pot three-component synthesis of trisubstituted imidazole derivatives using benzil or 9,10-phenanthrenequinone, different aldehydes, and ammonium acetate in EtOH solvent at 80 °C over 30 min. The yields of products were excellent, in the range 83–97%. The catalyst showed outstanding catalytic performance toward heating conditions and good reusability. Also, this methodology had several advantages, such as simple procedures, short reaction time, excellent yield, simple workup, and mild reaction conditions.

## Abstract 2

Cellulose is an eco-friendly, efficient, and suitable substrate for use as a coating material and support in the preparation of catalysts.

## Graphical abstract

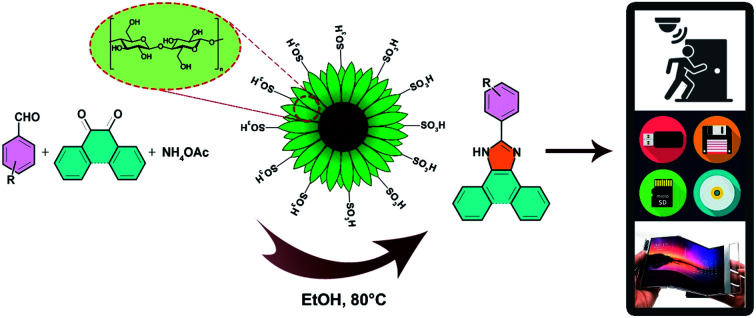

## 1. Introduction

Nowadays, green chemistry encourages scientists to minimize the harmful impacts of chemical processes on the environment by changing the design of chemical reactions and processes.^1^ Synthesis of organic compounds using multi-component reactions in the presence of a functionalized supported heterogeneous catalyst exactly accords with the green chemistry program.^2^

Heterogeneous catalysts have received significant attention because of their simple separation from the reaction mixture, reusability, and long-term stability.^3^ To produce a functionalized heterogeneous catalyst, a proper selection of support is vital due to increasing the performance and activity of the catalyst, improving its ability to stabilize nanoparticles and their durability, enhancing thermal stability, increasing the surface area and surface active sites, high catalyst dispersion, and effective contact with the substrates.^4,5^ Hence, various materials have been used as support such as silica,^6^ bio-based materials, insoluble polymers,^7^ and magnetic nanoparticles (MNPs).^8^ MNPs are very useful since they have large surface areas, can be simply synthesized and they can easily separate from the reaction mixture using an external strong magnet.^9,10^ Among the bio-based supports, cellulose-supported MNPs are a series of attractive heterogeneous catalysts because of the unique properties of cellulose such as sustainability, accessibility, low cost, easy handling, and eco-friendly nature. Moreover, the hydroxyl groups on cellulose can be easily functionalized by suitable reagents to produce more appropriate and active catalysts.^11^ Thus cellulose-supported MNPs are excellent candidates both from the perspective of green chemistry and a large number of functional groups.

Multi-component reactions (MCRs) are one of the most important types of chemical reactions owing to their valuable properties such as efficiency, safety, atom-economic chemistry, straightforward reaction design, and simultaneous combination of at least three reactants at a single vessel to selectively obtain the desired product.^12^ These reactions have many fundamental roles in medicinal chemistry,^13^ combinatorial chemistry, polymer chemistry,^14^ and synthesis of many useful and important small molecules, heterocyclic compounds and natural products.^15,16^

Imidazole is one of the most valuable five-membered heterocyclic compounds with two nitrogen atoms.^17^ This compound is found in the structure of various materials (*i.e.* composites,^18^ ionic liquids,^19^ metal-coordinating ligands^20^ host materials) and is widely applied in photonic and electronic areas such as OLEDs,^21^ optical sensors^22^ and memory devices^23^ and natural products.^17^ Besides, imidazole derivatives demonstrate a great variety of pharmaceutical and biological activities such as antitumor, anticancer, antimicrobial herbicidal, anti-inflammatory, and anti-diabetic.^24^ Various methods have been reported for the synthesis of imidazole.^25^ One of the best synthetic methods is three component reaction of a 1,2-dicarbonyl compound, aldehydes and ammonia. This reaction has been reported in the presence of various heterogeneous catalysts such as Co(ii) salen@KCC-1,^26^ heteropolyacid,^27^ molecular sieve supported titanium,^28^ NaHSO_4_–SiO_2_,^29^ NiFe_2_O_4_@SiO_2_@aminoglucose,^30^ Fe_3_O_4_@chitosan,^31^ clays, zeolite, nanocrystalline sulfated zirconia,^32^ chitosan–SO_3_H,^33^ in different conditions. However, most of them suffer from poor yields^34^ high temperatures,^35^ the presence of hazardous solvents or toxic metal catalysts,^36^ the existence of side reactions, and tedious and time-consuming multi-step procedures.^35^ Therefore, scientists are still trying to find eco-friendly ways to synthesize these valuable compounds in the presence of efficient catalysts under mild conditions.^37,38^

For this purpose, Fe_3_O_4_ nanoparticles were covered with cellulose fibers and the resulting nanocomposite was modified with chlorosulfonic acid to obtain MNP–cellulose–OSO_3_H (MC–SO_3_H) catalyst. The performance of this catalyst was evaluated in the synthesis of trisubstituted imidazoles and the effect of different parameters on the yield of the reaction was assayed. Also, catalyst reusability was investigated.

## 2. Experimental

### 2.1. Materials

Cellulose, iron(iii) chloride hexahydrate, iron(ii) chloride tetrahydrate, and chlorosulfonic acid were acquired from Merck. Other chemicals were purchased from other commercial resources and used without further purification.

### 2.2. Catalyst preparation

#### 2.2.1. Preparation of magnetic cellulose nanoparticles

For preparing magnetic cellulose (MC) nanoparticles, 3 g of cellulose was added to a solution of iron(iii) chloride hexahydrate (4.6 g, 0.017 mol), iron(ii) chloride tetrahydrate (2.2 g, 0.011 mol), 1 mL acetic acid and 100 mL distilled water into a 400 mL Erlenmeyer flask and stirred for 10 min at 100 °C until the solid was dissolved to some extent. The mixture was exposed to ultrasound radiation for 10 min at 40 °C under a nitrogen atmosphere and then 15 mL aqueous ammonia (25%) was added to the mixture. The obtained precipitates containing MC nanoparticles were removed using an external magnet, washed several times with distilled water, and dried in an oven at 60 °C for 24 h.

#### 2.2.2. Preparation of MC–OSO_3_H nanoparticles

**Scheme 1:**
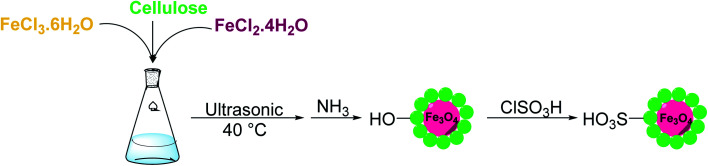
Synthesis steps for preparation of MC–OSO_3_H nanoparticles.

To prepare the modified MC, 0.5 mL of ClSO_3_H was added dropwise to a mixture of 2 g MC and 6 mL dry CHCl_3_ under vigorous stirring at 0 °C during 30 min. This mixture was allowed to stir at room temperature for another 1.5 h and then the sulfonated magnetic cellulose (MC–SO_3_H) nanoparticles were separated by an external magnet. Finally, these particles were washed multiple times with methanol and water, and dried in an oven at 60 °C for 24 h. The synthesis steps for the preparation of MC–OSO_3_H nanoparticles are illustrated in Scheme 1.

### 2.3. Characterization techniques

A Büchi B-545 apparatus was applied to determine the melting points in open capillary tubes. The ^1^H NMR spectra were recorded on a Bruker AVANCE DPX FT-NMR spectrometer at 400 MHz (*δ* in ppm). Inductively coupled plasma optical emission spectrometry (ICP-OES) was performed on a Varian Vista PRO Radial. FT-IR spectra were recorded on a JASCO FT/IR-6300 spectrometer using the KBr disk method at 400–4000 cm^−1^ at room temperature. Elemental analysis was used to estimate the elemental composition using a Heraeus CHNS Rapid analyzer. High angle powder X-ray diffraction (XRD) analysis was carried out on a Bruker D8 ADVANCE diffractometer, equipped with Ni-filtered Cu Kα (*K* = 0.15406 nm) radiation. The thermogravimetric analysis was performed by a SDT Q600 V20.9 Build 20 thermogravimetric system over a temperature range 25–700 °C at a scan rate of 5 °C min^−1^ in N_2_ atmosphere. A MIRA3 TESCAN-XMU microscope was applied to take FE-SEM, EDX, and elemental mapping images. Transmission electron microscopy (TEM) images were acquired on a Philips EM 208S apparatus. Zeta Potential measurements were performed using a SZ-100 nanoparticle analyzer at 25 °C. The magnetic properties of the particles were investigated by a vibrating sample magnetometer (LBKFB 1.5 tesla).

### 2.4. Typical experimental procedure for the synthesis of 2,4,5-trisubstituted imidazole (3a)

4 mL of ethanol was added to a mixture of 4-bromobenzaldehyde (0.185 g, 1 mmol), benzil (0.210 g, 1 mmol), ammonium acetate (0.231 g, 3 mmol), and MC–SO_3_H (0.012 g) in a reaction tube at 80 °C for 2 h. The progress of the reaction was monitored by thin-layer chromatography (TLC) using silica gel plates (SIL G/UV 254) and after completion of the reaction, the mixture was cooled to room temperature, filtered, and washed with EtOH/H_2_O (1 : 1 v/v) to obtain the pure product (3a) as a white solid (0.360 g, 96%, mp: 251–253 °C). The desired product was confirmed by H-NMR.

### 2.5. Investigation of acidity and reusability of the catalyst

The number of acidic sites (H^+^) of the MC–SO_3_H was determined by acid–base back titration. 10 mg of catalyst was added to 3 mL of 0.01 N NaOH solution and stirred for 1 h in an Erlenmeyer flask. Then the excess amount of base was titrated by the addition of 0.01 N HCl solution.^39^ Also, the reusability of the catalyst was evaluated in the model reaction under optimum conditions. After completion of the reaction, the catalyst was separated by an external magnet, washed with ethanol and acetone, and dried in an oven at 70 °C for 3 h before using it in a subsequent run. This procedure was repeated three more times and the product yield was obtained at the end of each cycle.

## 3. Results and discussion

### 3.1. Catalyst characterization

#### 3.1.1. FTIR analysis

**Fig. 1:**
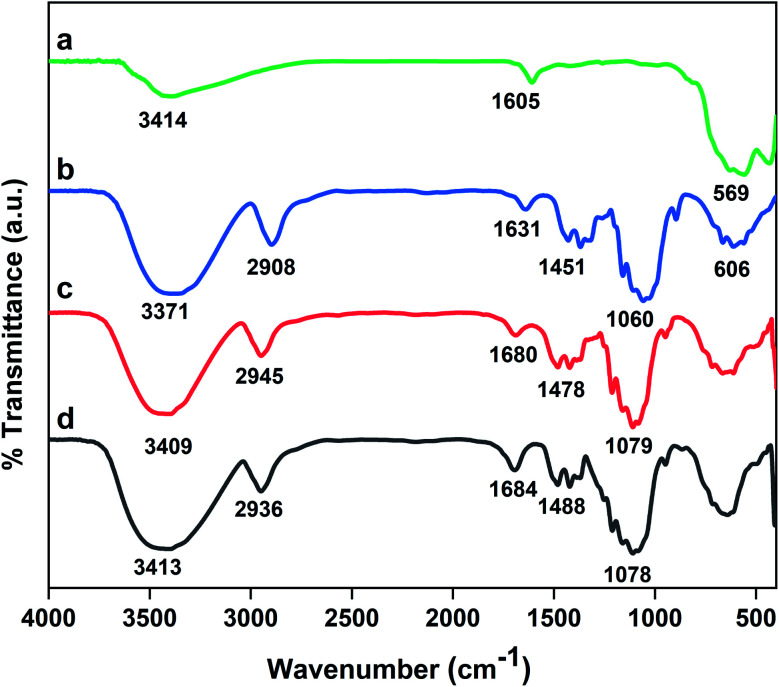
FT-IR spectra of (a) Fe_3_O_4_, (b) cellulose, (c) MC, (d) MC–SO_3_H.

The results of FT-IR analysis are presented in Fig. 1. The characteristic peak of Fe_3_O_4_ nanoparticles appears at 569 cm^−1^.^40^ The peak at 3414 cm^−1^ is assigned to the stretching vibrations of surface water molecules.^41^ The spectrum of cellulose shows a broad absorption band at 3371 cm^−1^ related to OH groups and a band at 2908 cm^−1^ corresponding to C–H stretching vibrations. Also, the strong band at around 1060 cm^−1^ is attributed to 1,4-β-glycosidic bonds of cellulosic units.^42^ In the FT-IR spectrum of MC, the absorption band related to O–Fe bonds should be observed at 569 cm^−1^ but it is covered with a wide peak of cellulose at about 600 cm^−1^. Also, in the case of MC–SO_3_H, two absorption bands at 1110 and 1161 cm^−1^ due to the asymmetric and symmetric stretching vibrations of O=S=O are overlapped with broad stretching 1,4-β-glycosidic bonds of cellulose.^43^ Notably, the peak corresponding to O–H bending of adsorbed water molecules on the surface is observed at 1605–1684 cm^−1^ in all spectrums.

#### 3.1.2. Elemental analysis and ICP

Elemental analysis was used to estimate the chemical compositions of MC and MC–SO_3_H samples. The results summarized in Table 1 confirm the successful sulfonation of cellulose due to the existence of sulphur in the structure of the MC–SO_3_H catalyst. According to this result, 0.6 mmol g^−1^ was the amount of acid loaded on the surface of the catalyst. Also, the amount of Fe in the structure of the MC–SO_3_H catalyst is very close to the used molar ratio in the synthesis of the MC–SO_3_H (17%), indicating the efficacy of the preparation procedure used in the present work.

**Table 1:** The results of elemental analysis of the samples

Sample	C (%)	H (%)	N (%)	S (%)	Fe^a^ (%)
MC	37.77	5.77	—	—	23
MC–SO_3_H	30.64	4.74	—	1.93	20

a Estimated by ICP-OES.

#### 3.1.3. XRD analysis

**Fig. 2:**
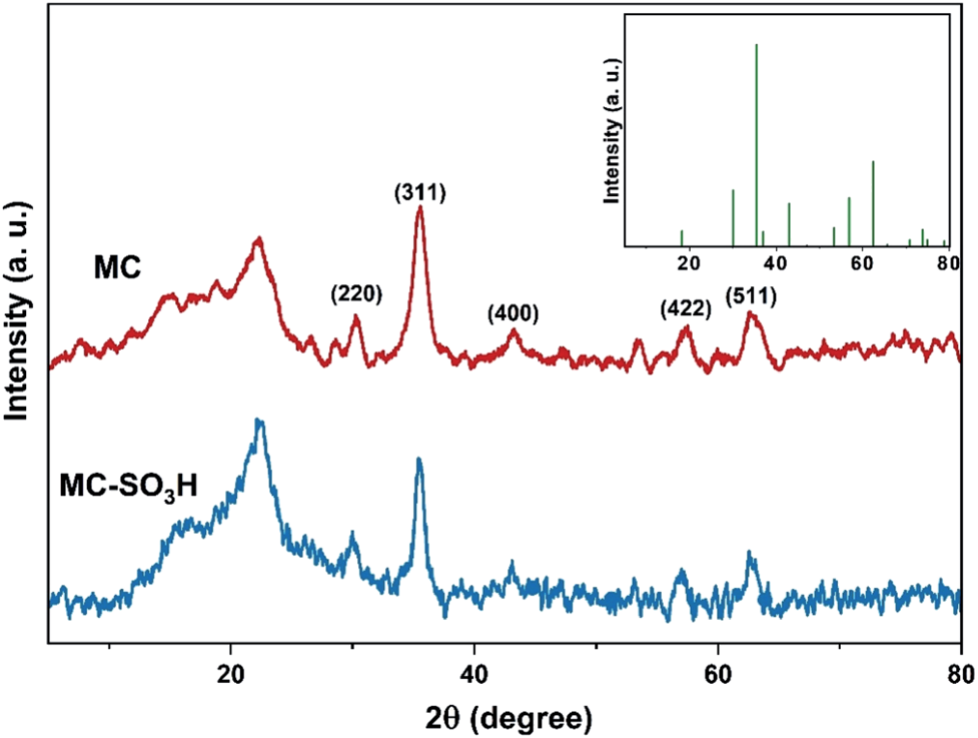
XRD patterns of MC and MC–SO_3_H (inset image: the standard pattern of Fe_3_O_4_).

The XRD patterns of MC and MC–SO_3_H are shown in Fig. 2. The powder XRD patterns of MC and MC–SO_3_H show two peaks at 2*θ* = 15.1 and 22.5° corresponding to cellulosic peaks,^44^ and the peaks at 2*θ* = 30.3, 35.6, 43.2, 53.5, 57.5 and 62.7° related to the (220), (311), (222), (400), (422) and (511) planes of Fe_3_O_4_, respectively. This observation demonstrates that coating of Fe_3_O_4_ nanoparticles with cellulose has not changed the crystalline construction of Fe_3_O_4_. These results clearly reveal the existence of Fe_3_O_4_ nanoparticles in the cellulosic matrix of MC and MC–SO_3_H.

#### 3.1.4. SEM, EDX and elemental mapping

**Fig. 3:**
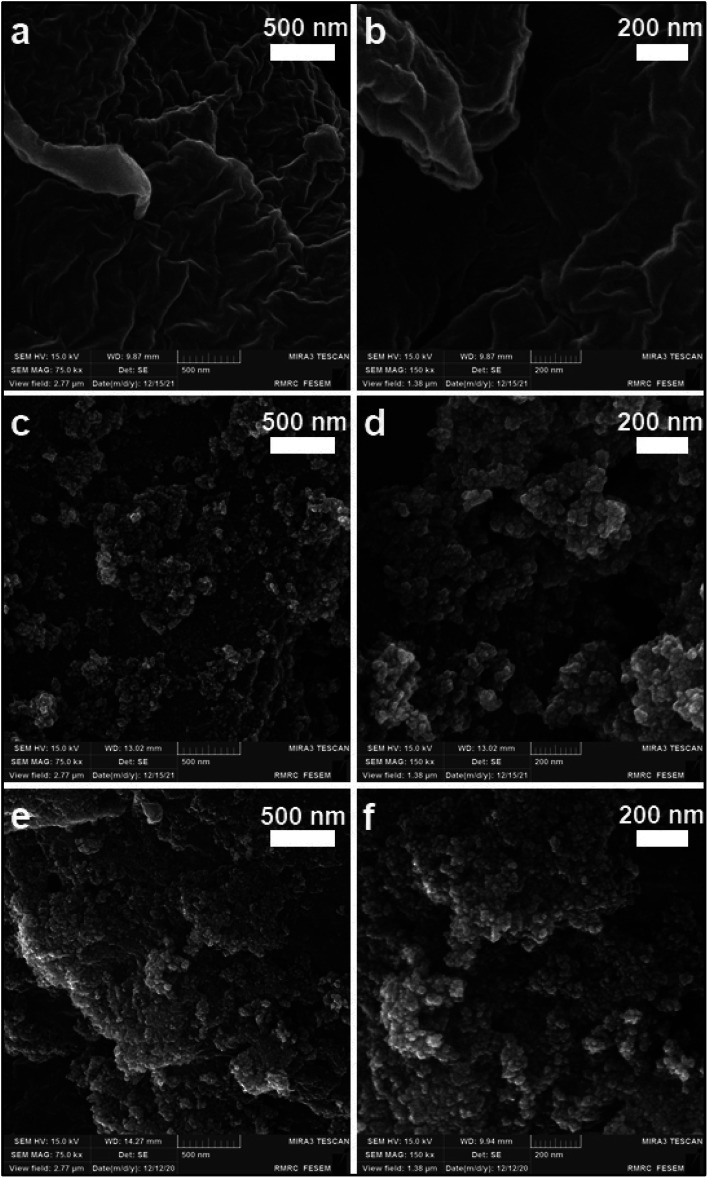
FE-SEM images of (a and b) cellulose, (c and d) MC and (e and f) MC–SO_3_H.

**Fig. 4:**
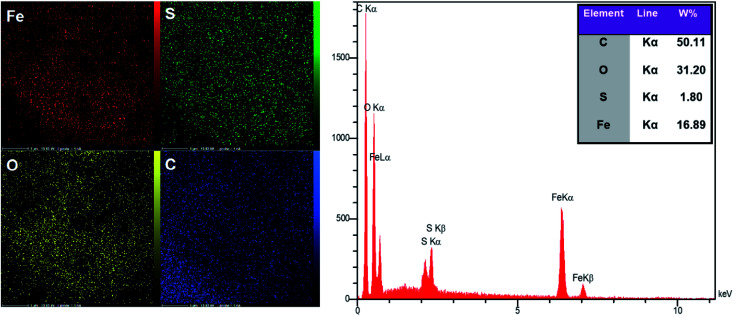
Elemental mapping and EDX images of MC–SO_3_H.

The morphology of cellulose, MC, and MC–SO_3_H were studied using field effect scanning electron microscopy (FE-SEM) as illustrated in Fig. 3. As can be observed, cellulose possesses a uniform and soft structure (Fig. 3a and b). In the MC FE-SEM images (Fig. 3c and d) the spherical magnetic nanoparticles coated with cellulose layer is clearly detectable. This structure confirms that Fe^3+^ and Fe^2+^ ions in the cellulose aqueous solution have been converted to magnetic nanoparticles. Due to the formation of nanoparticles in the confined empty spaces of cellulose fibers, the dimensions of the particles produced in this method are less than those of the magnetic nanoparticles obtained by fabrication and coating in the separated steps.^45^ The comparison of these images with MC–SO_3_H ones indicates that the modification step had not affected the morphology of MC. Also, energy dispersive X-ray (EDX) analysis and elemental mapping affirm the existence of the S element and the homogenous distribution of the participated elements on the surface of MC–SO_3_H (Fig. 4).

#### 3.1.5. TEM analysis

**Fig. 5:**
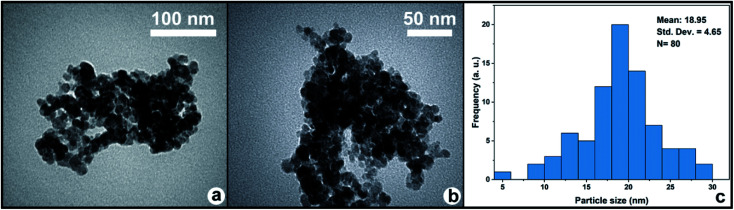
(a and b) TEM images of MC–SO_3_H, and (c) size distribution diagram of MC–SO_3_H.

Transmission electron microscopy (TEM) was used to investigate the morphological properties of MC–SO_3_H (Fig. 5a and b). As can be seen, these images demonstrate a narrow particle size distribution for the nanospheres of the catalyst with an approximately uniform shape. Further, MC–SO_3_Hs had a narrow particle size distribution ranging from 7 to 30 nm and the average size of MC–SO_3_H was found to be 18.95 nm (Fig. 5c).

#### 3.1.6. Zeta potential analysis

**Fig. 6:**
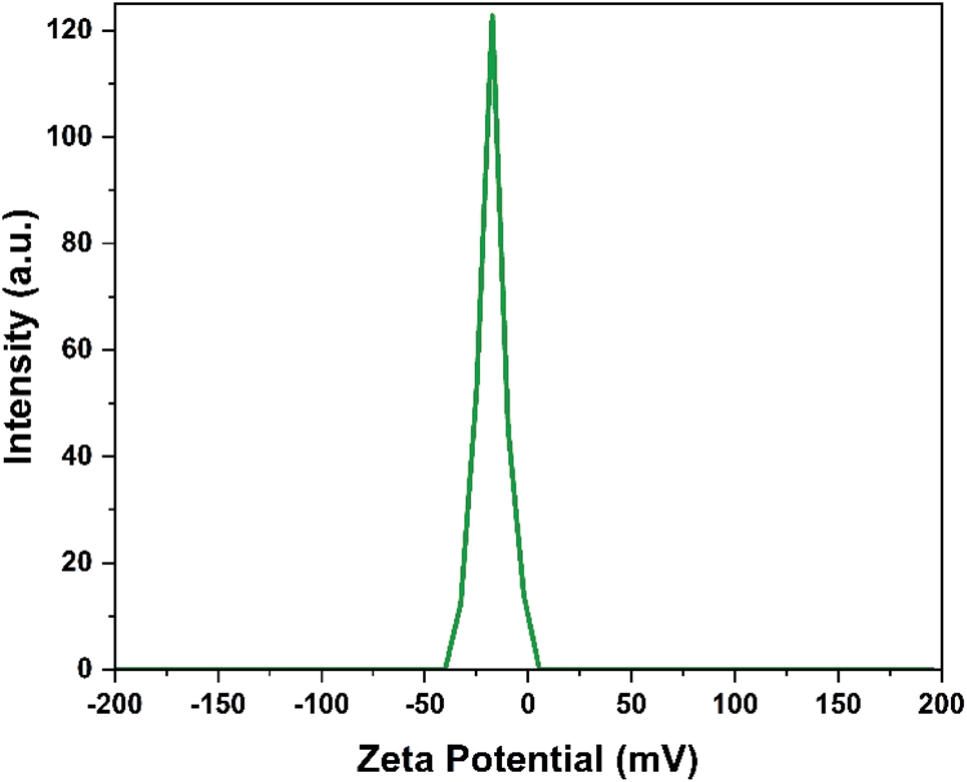
Zeta potential analysis of MC–SO_3_H.

The interfacial layer charge of MC–SO_3_H was evaluated by zeta potential analysis and the result is illustrated in Fig. 6. From the result, the zeta potential of MC–SO_3_H measured in the aqueous medium was found to be −17 mV. This negative value shows the presence of the electronegative functional groups formed by the deprotonation of the SO_3_H groups at the surface of the catalyst. This observation clearly confirms the existence of the SO_3_H groups in the structure of the MC–SO_3_H catalyst.

#### 3.1.7. VSM analysis

**Fig. 7:**
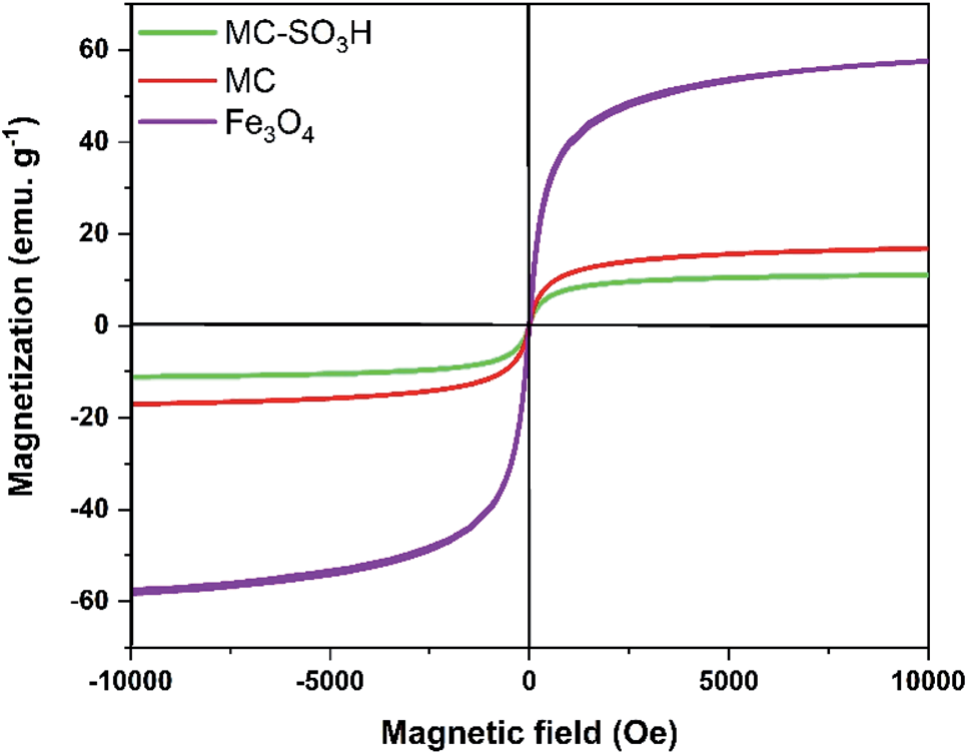
Magnetization curves for Fe_3_O_4_, MC and MC–SO_3_H at room temperature.

The magnetic features of MC–SO_3_H and MC were accomplished by a vibrating sample magnetometer (VSM) at room temperature to assay the loading of various functional groups on MNPs (Fig. 7). On the basis of Fig. 7, the values of saturation magnetization (*M*_s_) for MC and MC–SO_3_H were measured at 16.93 and 11.30 emu g^−1^, respectively. The difference between these saturation magnetization values can be attributed to the being quenched of the surface moments due to the conversion of the –OH functional groups to –SO_3_H around the MC cores because the substituted –SO_3_H groups occupy more volume and create more physical distance between particles. The strong magnetization of the nanoparticles was confirmed by simple attraction with an external magnet. Accordingly, the obtained magnetic nanoparticles possessed excellent magnetic properties which suggest that they can prevent from aggregating and enable to redisperse rapidly when the magnetic field is removed. This result also implies that the functionalized magnetic nanoparticles have a lower magnetic quantity relative to bar magnetic nanoparticles (59.19 emu g^−1^).

#### 3.1.8. Thermogravimetric analysis (TGA) and acidity

**Fig. 8:**
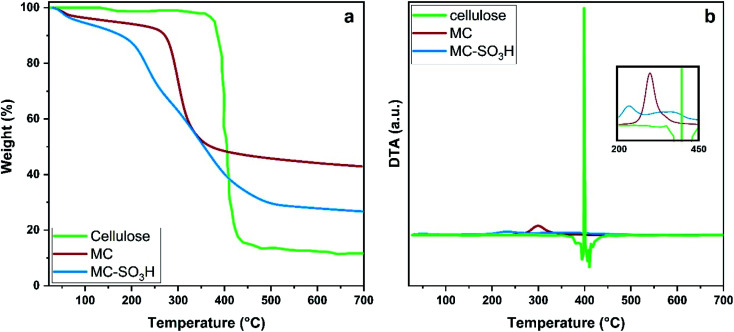
(a) TGA and (b) DTA thermograms of cellulose, MC and MC–SO_3_H.

Fig. 8a represents the thermal gravimetric analysis diagrams to evaluate the thermal stability of cellulose, MC, and MC–SO_3_H by increasing the temperature from 25 to 700 °C. Bare Fe_3_O_4_ thermal stability is superb and its weight loss is about 2% over the temperature range of 25 to 700 °C.^46^ According to the TGA curves, the weight losses of cellulose, MC and MC–SO_3_H are about 87%, 57%, and 71%, respectively. The thermogram of cellulose shows a two-stage decomposition: one at about 120 °C and another at 355–480 °C. The first weight loss of 3% is due to the loss of adsorbed water molecules, and the second weight loss of 85% is related to cellulose degradation. Also, the weight loss of MC has occurred in two stages, the first is related to the loss of solvents and water molecules from the surface of the catalyst about 6% below 110 °C, and the numerous weight losses in the second step is attributed to disintegration of organic polysaccharide layer at 233–400 °C (about 45%).^47^ This thermal resistance reduction confirms the modification performed on cellulose. The curve of MC–SO_3_H indicates three steps with the weight loss of 7%, 21%, and 42%, respectively. The considerable weight losses between 153–267 and 267–550 °C are attributed to the sulfonic acid and organic moieties grafted to the surface of Fe_3_O_4_ MNPs. According to this analysis, it was found that 2.53 mmol g^−1^ of SO_3_H groups were loaded in the structure of MC–SO_3_H catalyst which is higher than the actual level of acidity due to the destruction of the polysaccharide units along with the SO_3_H groups and this result is almost in agreement with the results of back titration and CHNS (Table 2).

**Table 2:** Acidity of MC–SO_3_H catalyst obtained by different methods

Method	H^+^ content (mmol g^−1^)
CHNS	0.6
Back titration	1.02
TGA	2.53

### 3.2. Optimization of reaction conditions

In order to investigate the catalytic activity, MC–SO_3_H catalyst was used in a one-pot three-component reaction for the synthesis of trisubstituted imidazoles. For this purpose, 4-bromobenzaldehyde, benzil and ammonium acetate were selected as starting materials for the model reaction in the presence of MC–SO_3_H. Since the polarity of the solvent may be effective in this reaction, various solvents with different polarities were tested. The results shown in Table 3 indicate that the maximum yield of the product was obtained in the presence of ethanol.

**Table 3:** Effect of different solvents on the model reaction^a^

Entry	Solvent	Temperature (°C)	Yield^b^ (%)
1	Water	80	79
2	Methanol	Reflux	84
3	Ethanol	80	96
4	Propanol	80	67
5	Dioxane	80	28
6	THF	Reflux	16
7	Acetonitrile	Reflux	38
8	Ethyl acetate	Reflux	41
9	Chloroform	Reflux	N.R
10	Solvent free	80	N.R

a Reaction conditions: 4-bromobenzaldehyde (1 mmol), benzil (1 mmol), ammonium acetate (3 mmol), MC–SO_3_H (20 mg), solvent (4 mL), 2 h.

b Isolated yield.

**Scheme 2:**
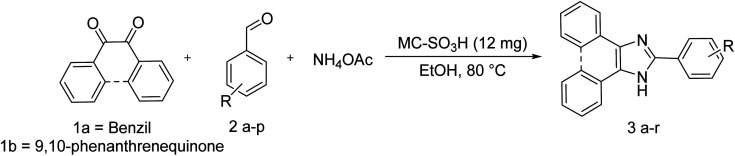
Synthesis of compound 3 using benzil or 9,10-phenanthrenequinone, different aldehydes and ammonium acetate under optimized reaction conditions.

In the next step, a series of experiments were performed to investigate the effect of the reaction temperature, amount of catalyst, and reaction time on the yield of the product and the results are reported in Table 4. Temperature is one of the most important factors affecting the yield of reactions. To evaluate the importance of temperature in the yield of the reaction, the model reaction was carried out under thermal condition at different temperatures, and it was observed that the temperature of 80 °C as the optimal temperature gives the best yield (Table 4, entries 1–3). The results of the experiments related to the investigation of the catalyst amount indicated that with increasing the amount of catalyst from 5 to 12 mg (Table 4, entries 4–6), the yield of the reaction increased from 37 to 96%. However, the further increase in the catalyst amount did not change the reaction yield (Table 4, entry 7). Finally, after optimization of solvent, temperature, and catalyst amount, the model reaction was carried out in different time periods (Table 4, entries 8–11). As can be seen in Table 4, the highest yield of the product was obtained in about 30 minutes and a further increase in reaction time had no effect on the product yield (Scheme 2). Finally, various derivatives of the product were synthesized under optimum reaction conditions in the presence of MC–SO_3_H catalyst (Table 5). To demonstrate the efficiency and repeatability of the intended catalyst, a wide variety of aldehydes with acceptor and donor substitutions were used to synthesis different trisubstituted imidazoles. The results exhibited that the product yields have not significantly influenced by various substituents of the aldehydes and all the products were acquired in high yields.^31,48^

**Table 4:** The results of optimization of reaction conditions on the model reaction^a^

Entry	Amount of MC–SO_3_H (mg)	Temperature (°C)	Time (min)	Yield^b^ (%)
1	20	R.T	120	Trace
2	20	50	120	62
3	20	80	120	96
4	5	80	120	37
5	10	80	120	79
6	12	80	120	96
7	15	80	120	96
8	12	80	60	96
9	12	80	30	96
10	12	80	20	90
11	12	80	10	69

a Reaction conditions: 4-bromobenzaldehyde (1 mmol), benzil (1 mmol), ammonium acetate (3 mmol), EtOH (4 mL).

b Isolated yield.

**Table 5:** One-pot synthesis of trisubstituted imidazoles in the presence of MC–SO_3_H catalyst^a^

Comp.	α-Diketone	R	Yield (%)	Mp (°C)	Ref.
3a	1a	4-Br–C_6_H_4_	96	251–253	249–251 (ref. 49)
3b	1a	4-F–C_6_H_4_	95	187–189	189–190 (ref. 50)
3c	1a	3-F–C_6_H_4_	91	>260	284–285 (ref. 50)
3d	1a	2,6-diCl–C_6_H_4_	83	231–232	229–230 (ref. 51)
3e	1a	4-CN–C_6_H_4_	97	>260	259–261 (ref. 49)
3f	1a	3-NO_2_–C_6_H_4_	90	>260	301–303 (ref. 49)
3g	1a	4-OMe–C_6_H_4_	89	222–224	219–221 (ref. 49)
3h	1a	2-Thiophene	87	254–256	257–259 (ref. 49)
3i	1b	C_6_H_4_	89	>260	311–313 (ref. 52)
3j	1b	4-Br–C_6_H_4_	95	>260	280–282 (ref. 52)
3k	1b	4-Cl–C_6_H_4_	93	>260	274–276 (ref. 52)
3l	1b	2-Cl–6-F–C_6_H_4_	87	>260	—
3m	1b	4-NO_2_–C_6_H_4_	93	>260	335–337 (ref. 52)
3n	1b	2-NO_2_–C_6_H_4_	94	>260	267 (ref. 53)
3o	1b	4-iPr–C_6_H_4_	90	>260	—
3p	1b	4-Ph–C_6_H_4_	94	>260	258–259 (ref. 54)
3q	1b	2-OH–5-NO_2_–C_6_H_4_	88	>260	259–260 (ref. 55)
3r	1b	4-OMe–C_6_H_4_	91	252–254	254–255 (ref. 56)

a Reaction conditions: 4-bromobenzaldehyde (1 mmol), benzil (1 mmol), ammonium acetate (3 mmol), EtOH (4 mL).

**Scheme 3:**
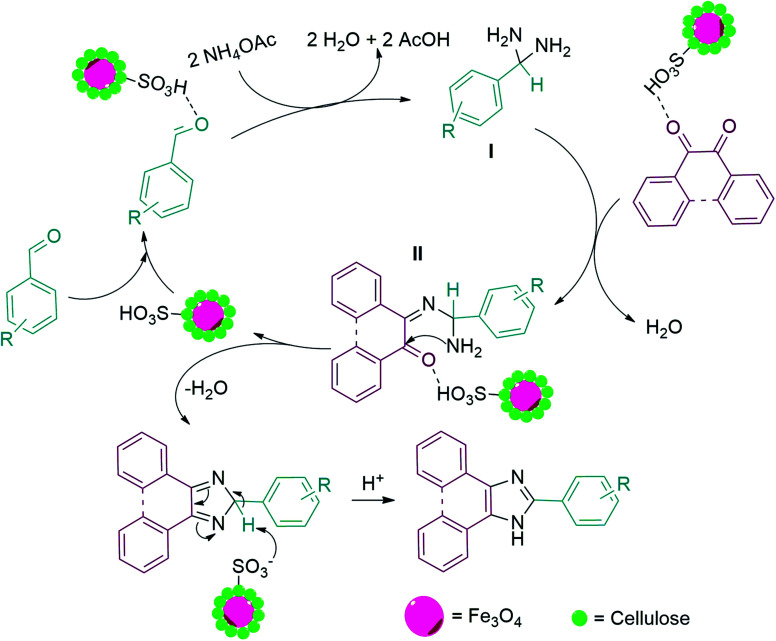
Suggested mechanism for the synthesis of 2,4,5-trisubstituted imidazole derivatives in the presence of MC–SO_3_H catalyst.

The proposed mechanism for the synthesis of trisubstituted imidazole is displayed in Scheme 3. As can be found, the carbonyl group of the aldehyde was firstly activated by MC–SO_3_H catalyst and attacked by two ammonia molecules (produced from the decomposition of ammonium acetate) that formed diamine I. In the following, one of the carbonyl groups of benzil, activated by MC–SO_3_H catalyst reacted by one amine groups of the diamine intermediate I which resulted in intermediate II. Then, the second amine group attacked to other carbonyl *via* intermolecular cyclization reaction. In the final stage, trisubstituted imidazole was produced by elimination of a H_2_O molecule.^57^

### 3.3. Catalyst reusability study

**Fig. 9:**
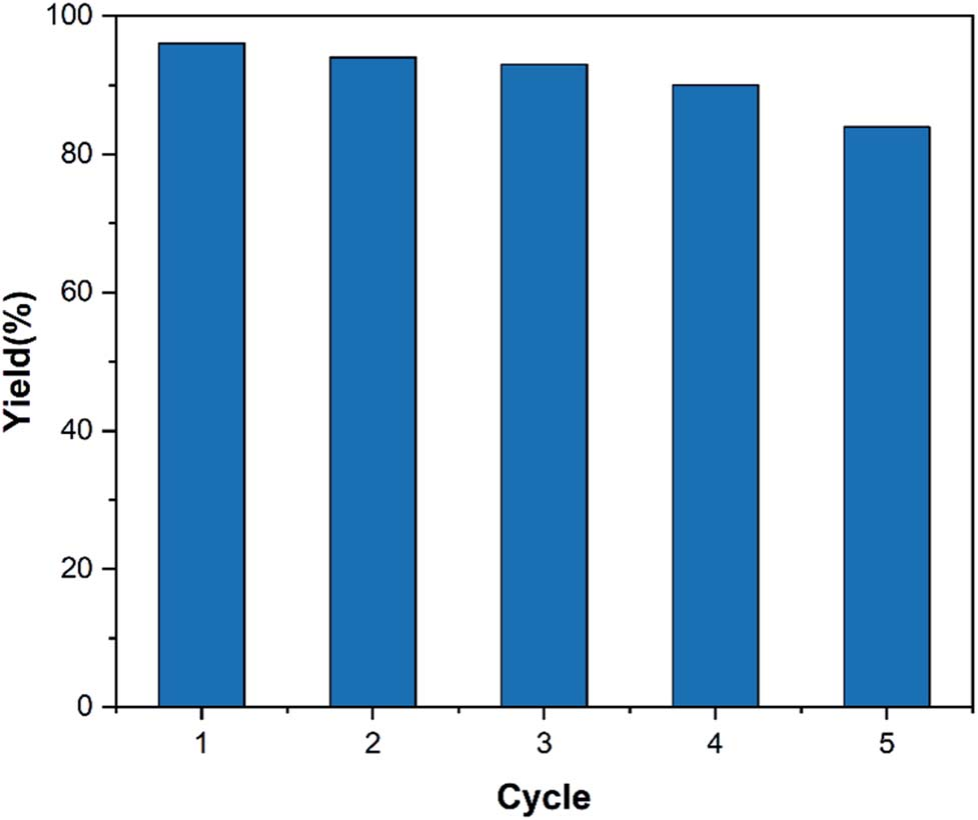
Reusability of the MC–SO_3_H catalyst for the model reaction.

**Fig. 10:**
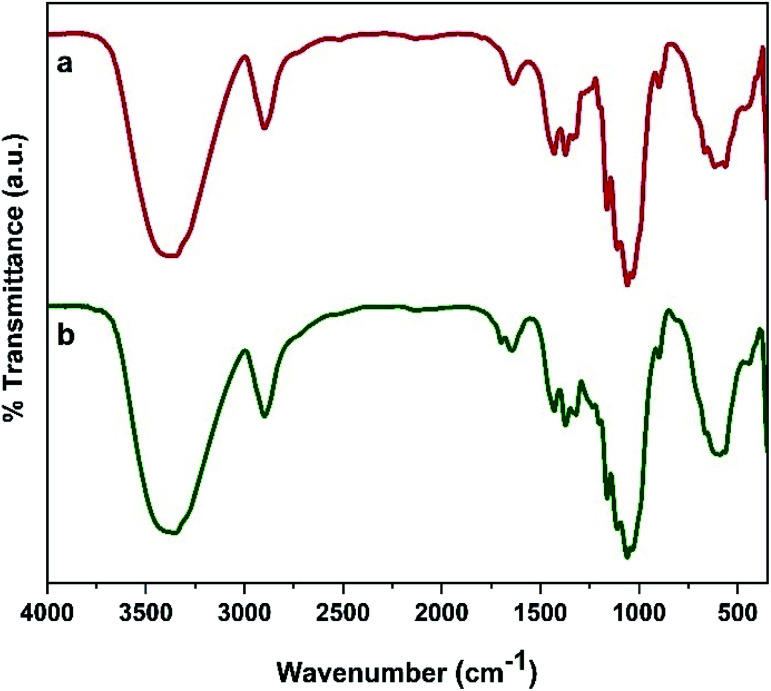
FT-IR spectra of (a) fresh catalyst, (b) five cycles recovered catalyst and SEM images of catalyst after five rounds.

The reusability of the catalyst is one of the main advantages of using a heterogeneous catalyst. Therefore, this parameter was investigated according to the procedure mentioned in Section 2.5. The results displayed in Fig. 9 show that the decrease in product yield is about, probably due to the breakdown of some –OSO_3_H bonds on the surface of nanocomposite particles which leads to reduce the number of –OSO_3_H agents on the surface of the catalyst and reduction of catalyst efficiency. Characterization of the recovered catalyst was done with FT-IR spectroscopy and SEM analysis. The SEM images of the recovered catalyst (Fig. 10) exhibited that the morphology of the catalyst was conserved during its reuse.

### 3.4. Comparison of this catalyst with other heterogeneous catalysts

Since the imidazoles are one of the most interesting compounds, various heterogeneous catalysts were introduced to synthesize these derivatives. Here, the catalytic performance of MC–SO_3_H catalyst was compared to that of reported heterogeneous catalysts for the synthesis of 2,4,5-trisubstituted imidazole derivatives and the results are presented in Table 6. Approximately, most of these catalysts have some problems such as intricate preparations, high cost, long time, and complicated recycling. For example, Shen *et al.* synthesized these derivatives in the presence of Yb(OPf)_3_ catalyst (6.7 mg) and perfluorodecalin (1.5 mL) in AcOH at 80 °C for a long period time (360 minutes) with 85% yield. This catalyst was recovered and reused for 5 cycles. In another study, Magyar *et al.* reported a time-consuming method by using toluene, which is a toxic, non-green organic solvent at 100 °C, in the presence of 100 mg catalyst (Ti^4+^/4 Å MS) with a yield of 85%. Also, Arghan *et al.* investigated the synthesis of 2,4,5-trisubstituted imidazole derivatives in solvent free condition at 100 °C, in presence of 15 mg Fe_3_O_4_/PVAm–SO_3_H catalyst. The yield of this reaction was 89% and the catalyst was recovered and reused for 8 cycles.

**Table 6:** Catalytic performance of various heterogeneous catalysts for the synthesis of 2,4,5-trisubstituted imidazole derivatives

Catalyst (amount)	Reaction conditions	Time (min)	Run	Yield (%)	Ref.
Co(ii) salen complex@KCC-1 (4 mg)	EtOH, 80 °C	15	8	80	26
Yb(OPf)_3_ (6.7 mg), perfluorodecalin (1.5 mL)	AcOH, 80 °C	360	5	85	58
PMO-ICS (20 mg)	EtOH, reflux	42	4	98	59
Ti^4+^/4 Å MS (100 mg)	Toluene, 100 °C	660	4	85	28
Montmorillonite K10 (25 mg)	EtOH, reflux	110	3	75	32
Zeolite (25 mg)	EtOH, reflux	90	3	75	32
Nano-crystalline SZ (25 mg)	EtOH, reflux	75	3	81	32
Fe_3_O_4_/PVAm–SO_3_H (15 mg)	Solvent free, 100 °C	30	8	89	60
MC–SO_3_H (12 mg)	EtOH, 80 °C	30	5	96	This work

## 4. Conclusion

In the present work, MC–SO_3_H nanoparticles as a simple, non-expensive, and eco-friendly heterogeneous catalyst were prepared using cellulose as abundant biopolymer support. This catalyst was characterized by different methods and it was found that the modification approach was successfully performed. Then, MC–SO_3_H nanoparticles were used to synthesize a series of 2,4,5-trisubstituted imidazoles in EtOH solvent at 80 °C and the products were obtained in a short time with excellent yields. More importantly, these products were synthesized in the absence of hazardous organic solvents and under mild reaction conditions. Moreover, MC–SO_3_H nanoparticles were easily removed from the reaction mixture and reused for four cycles.

## Conflicts of interest

There are no conflicts to declare.

## Supplementary Material

RA-012-D2RA01348G-s001

## References

[cit1] Tang S. L. Y., Smith R. L., Poliakoff M. (2005). Green Chem..

[cit2] Tan J., Liu X., Yao N., Hu Y. L., Li X. H. (2019). ChemistrySelect.

[cit3] Dabiri M., Salehi P., Baghbanzadeh M., Zolfigol M. A., Agheb M., Heydari S. (2008). Catal. Commun..

[cit4] Yao N., Wu Y. P., Zheng K. B., Hu Y. L. (2018). Curr. Org. Chem..

[cit5] Wang G., Li F., Li L., Zhao J., Ruan X., Ding W., Cai J., Lu A., Pei Y. (2020). ACS Omega.

[cit6] Shamsa F., Motavalizadehkakhky A., Zhiani R., Mehrzad J., Hosseiny M. S. (2021). RSC Adv..

[cit7] Ren Y., Li H., Yang W., Shi D., Wu Q., Zhao Y., Feng C., Liu H., Jiao Q. (2019). Ind. Eng. Chem. Res..

[cit8] Zhou K., Liu X. P., Guo H., Li H. Q., Yang P. (2022). RSC Adv..

[cit9] Fekri L. Z., Pour K. H., Zeinali S. (2020). J. Organomet. Chem..

[cit10] Nikpassand M., Fekri L. Z., Nabatzadeh M. (2017). Comb. Chem. High Throughput Screening.

[cit11] Nordin A. H., Wong S., Ngadi N., Zainol M. M., Abd Latif N. A. F., Nabgan W. (2021). J. Environ. Chem. Eng..

[cit12] Fekri L. Z., Zeinali S. (2020). Appl. Organomet. Chem..

[cit13] Ruijter E., Orru R., Lam K., Timmerman H. (2018). Drug Discovery Today: Technol..

[cit14] Kakuchi R. (2019). Polym. J..

[cit15] Yao N., Lu M., Liu X. B., Tan J., Hu Y. L. (2018). J. Mol. Liq..

[cit16] Tan J., Li J. R., Hu Y. L. (2020). J. Saudi Chem. Soc..

[cit17] Verma A., Joshi S., Singh D. (2013). J. Chem..

[cit18] Mizuno M., Iwasaki A., Umiyama T., Ohashi R., Ida T. (2014). Macromolecules.

[cit19] Iijima G., Kitagawa T., Katayama A., Inomata T., Yamaguchi H., Suzuki K., Hirata K., Hijikata Y., Ito M., Masuda H. (2018). ACS Catal..

[cit20] Solomatina A. I., Chelushkin P. S., Abakumova T. O., Zhemkov V. A., Kim M., Bezprozvanny I., Gurzhiy V. V., Melnikov A. S., Anufrikov Y. A., Koshevoy I. O., Su S. H. (2018). Inorg. Chem..

[cit21] Yi R. H., Shao C. M., Lin C. H., Fang Y. C., Shen H. L., Lu C. W., Wang K. Y., Chang C. H., Chen L. Y., Chang Y. H. (2020). J. Phys. Chem. C.

[cit22] Wang J., Li R., Long X., Li Z. (2016). Sens. Actuators, B.

[cit23] Zhao K., Yu F., Liu W., Huang Y., Said A. A., Li Y., Zhang Q. (2019). J. Org. Chem..

[cit24] Jourshari M. S., Mamaghani M., Shirini F., Tabatabaeian K., Rassa M., Langari H. (2013). Chin. Chem. Lett..

[cit25] Hossain M., Nanda A. K. (2018). Science.

[cit26] Allahresani A., Naghdi E., Nasseri M. A. (2020). Inorg. Chem. Commun..

[cit27] Heravi M. M., Derikvand F., Bamoharram F. F. (2007). J. Mol. Catal. A: Chem..

[cit28] Magyar Á., Hell Z. (2019). Synlett.

[cit29] Karimi A. R., Alimohammadi Z., Azizian J., Mohammadi A. A., Mohammadizadeh M. R. (2006). Catal. Commun..

[cit30] Fekri L. Z., Nikpassand M., Shariati S., Aghazadeh B., Zarkeshvari R., Norouz N. (2018). J. Organomet. Chem..

[cit31] Zarnegar Z., Safari J. (2014). RSC Adv..

[cit32] Teimouri A., Chermahini A. N. (2011). J. Mol. Catal. A: Chem..

[cit33] Khan K., Siddiqui Z. N. (2015). Ind. Eng. Chem. Res..

[cit34] Singh I., Rani R., Luxami V., Paul K. (2019). Eur. J. Med. Chem..

[cit35] Wu X. J., Jiang R., Xu X. P., Su X. M., Lu W. H., Ji S. J. (2010). J. Comb. Chem..

[cit36] Salfeena C. T. F., Jalaja R., Davis R., Suresh E., Somappa S. B. (2018). ACS Omega.

[cit37] Zare Fekri L., Nateghi-Sabet M. (2021). J. Chin. Chem. Soc..

[cit38] Zare Fekri L., Nateghi M. (2021). Chem. Rev. Lett..

[cit39] Mohammadbagheri Z., Chermahini A. N. (2019). Chem. Eng. J..

[cit40] Huang H., Wang X., Ge H., Xu M. (2016). ACS Sustainable Chem. Eng..

[cit41] Sie Y., Wan C., Wu A. (2016). RSC Adv..

[cit42] Mohammadbagheri Z., Rahmati A., Hoshyarmanesh P. (2021). Int. J. Biol. Macromol..

[cit43] Chermahini A. N., Shahangi F., Dabbagh H. A., Saraji M. (2016). RSC Adv..

[cit44] Gong J., Li J., Xu J., Xiang Z., Mo L. (2017). RSC Adv..

[cit45] Yang Y., Zhang W., Yang F., Zhou B., Zeng D., Zhang N., Zhao G., Hao S., Zhang X. (2018). Nanoscale.

[cit46] Zhang Q., Han X., Tang B. (2013). RSC Adv..

[cit47] Azizi A. (2020). J. Inorg. Organomet. Polym. Mater..

[cit48] Naeimi H., Aghaseyedkarimi D. (2015). New J. Chem..

[cit49] Das B., Kashanna J., Kumar R. A., Jangili P. (2013). Monatsh. Chem..

[cit50] Jayram J., Jeena V. (2017). Green Chem..

[cit51] Xu F., Wang N., Tian Y., Li G. (2013). J. Heterocycl. Chem..

[cit52] Higuera N. L., Peña-Solórzano D., Ochoa-Puentes C. (2019). Synlett.

[cit53] Cook A. H., Jones D. G. (1941). J. Chem. Soc..

[cit54] Neunhoeffer O., Krieg G., Krieg B. (1966). Z. Naturforsch. B.

[cit55] Eseola A. O., Akogun O., Görls H., Atolani O., Kolawole G. A., Plass W. (2014). J. Mol. Catal. A: Chem..

[cit56] Eshghi H., Rahimizadeh M., Hasanpour M., Bakavoli M. (2015). Res. Chem. Intermed..

[cit57] Kanaani E., Nasr-Esfahani M. (2019). J. Chin. Chem. Soc..

[cit58] Shen M. G., Cai C., Yi W. B. (2008). J. Fluorine Chem..

[cit59] Dekamin M. G., Arefi E., Yaghoubi A. (2016). RSC Adv..

[cit60] Arghan M., Koukabi N., Kolvari E. (2019). J. Iran. Chem. Soc..

