# Supplementary material for: MNP–cellulose–OSO3H as an efficient and biodegradable heterogeneous catalyst for green synthesis of trisubstituted imidazoles
Source: RSC Adv. 2022 Apr 19;12(19):11740–9. doi: 10.1039/d2ra01348g (PMC9016742; doi:10.1039/d2ra01348g)
Supplement: RA-012-D2RA01348G-s001 [file RA-012-D2RA01348G-s001.pdf]

Electronic Supplementary Material (ESI) for RSC Advances.
This journal is © The Royal Society of Chemistry 2022

Supporting Information

In this supplementary material,

1. Characterization data of the compounds (**3a-3r**)
2. ^{1}H NMR of the compounds (**3a-3r**)
3. References

1. Characterization data of the compounds

2-(4-bromophenyl)-4,5-diphenyl-1H-imidazole (3a)

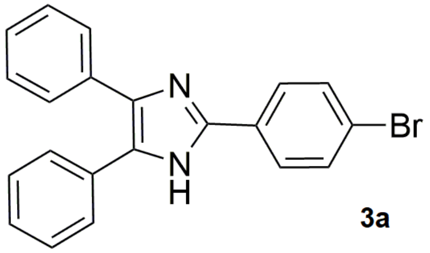

3a

Mp: 251-253 °C (249-251 °C)[1]; ^{1}H NMR (400 MHz, DMSO) δ 12.80 (s, 1H), 8.04 (d, *J* = 8.5. Hz, 2H), 7.70 (d, *J* = 8.6 Hz, 2H), 7.54 – 7.24 (m, 10H).

2-(4-fluorophenyl)-4,5-diphenyl-1H-imidazole (3b)

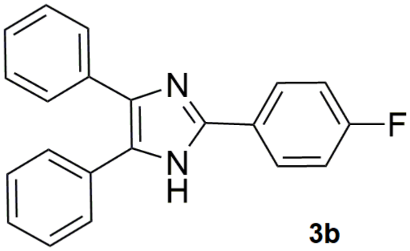

3b

Mp: 187-189 °C (189-190 °C)[2]; ^{1}H NMR (400 MHz, DMSO) δ 12.83 (s, 1H), 8.25 (d, *J* = 7.4, 2H), 8.23–7.32 (m, 12H).

2-(3-fluorophenyl)-4,5-diphenyl-1H-imidazole (3c)

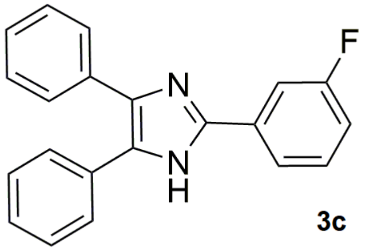

3c

Mp: >260 °C (284-285 °C)[2]; ^{1}H NMR (400 MHz, DMSO) δ 12.81 (s, 1H), 7.94 (d, *J* = 7.9 Hz, 1H), 7.88 (td, *J* = 10.0, 2.3 Hz, 1H), 7.59 9 – 7.37 (m, 8H), 7.32 (t, *J* = 7.3 Hz, 2H), 7.29 – 7.18 (m, 2H).

2-(2,6-dichlorophenyl)-4,5-diphenyl-1H-imidazole (3d)

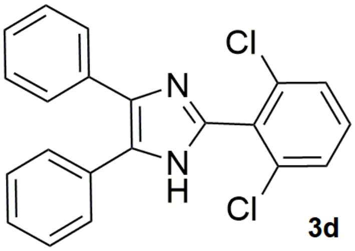

3d

Mp: 231-232 °C (229-230 °C)[3]; ^{1}H NMR (400 MHz, DMSO) δ 12.78 (s, 1H), 7.69 – 7.63 (m, 2H), 7.61 – 7.52 (m, 3H), 7.51 – 7.40 (m, 4H), 7.39 – 7.28 (m, 3H), 7.24 (t, *J* = 7.3 Hz, 1H).

4-(4,5-diphenyl-1H-imidazol-2-yl)benzonitrile (3e)

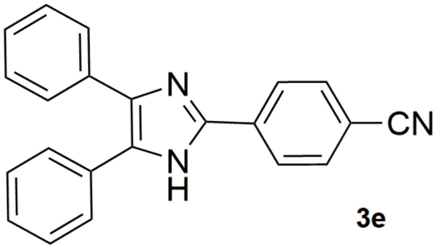

3e

Mp: >260 °C (259-261 °C)[1]; ^{1}H NMR (400 MHz, DMSO) δ 13.04 (s, 1H), 8.26 (d, *J* = 8.4 Hz, 2H), 7.96 (d, *J* = 8.4 Hz, 2H), 7.60 – 7.22 (m, 10H).

2-(3-nitrophenyl)-4,5-diphenyl-1H-imidazole (3f)

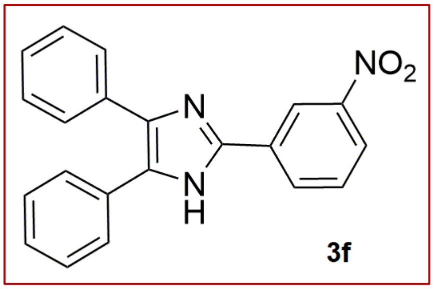

3f

Mp: >260 °C (301-303 °C)[1]; ^{1}H NMR (400 MHz, DMSO) δ 13.81 (s, 1H), 9.15 (s, 1H), 8.87 (s, 2H), 8.60 (s, 1H), 8.56 (s, 1H), 8.32 (dd, *J* = 8.1, 1.3 Hz, 1H), 7.91 (s, 1H), 7.87 (t, *J* = 8.0 Hz, 1H), 7.69 (s, 2H), 7.67 (m, 2H).

2-(4-methoxyphenyl)-4,5-diphenyl-1H-imidazole (3g)

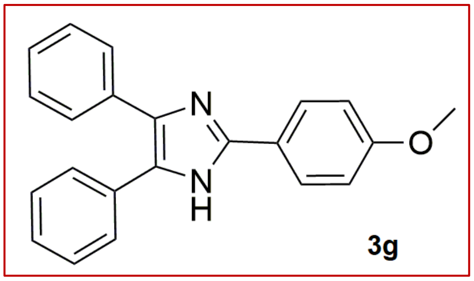

3g

Mp: 222-224 °C (219-221 °C)[1]; ^{1}H NMR (400 MHz, DMSO) δ 12.44 (s, 1H), 7.93 (d, *J* = 8.9 Hz, 2H),7.45 (d, *J* = 7.1 Hz, 2H), 7.41 (d, *J* = 7.0 Hz, 2H), 7.35 (t, *J* = 7.2 Hz, 2H), 7.27 (t, *J* = 7.3 Hz, 1H), 7.21 (t, *J* = 7.2 Hz, 2H), 7.13 (t, *J* = 7.3 Hz, 1H), 6.96 (d, *J* = 8.9 Hz, 2H), 3.73 (s, 3H).

4,5-diphenyl-2-(thiophen-2-yl)-1H-imidazole (3h)

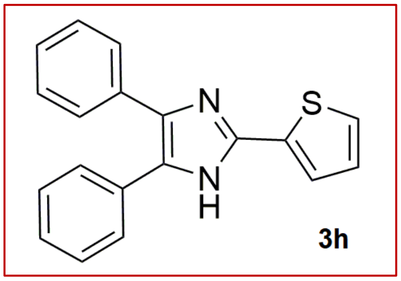

3h

Mp: 254-256 °C (257-259 °C)[1]; ^{1}H NMR (400 MHz, DMSO) δ 12.79 (s, 1H), 7.69 (dd, *J* = 3.6, 1.0 Hz, 1H), 7.57 (dd, *J* = 5.1, 1.0 Hz, 1H), 7.54 – 7.42 (m, 5H), 7.39 (tt, *J* = 3.1, 1.5 Hz, 1H), 7.31 (t, *J* = 7.2, 2H), 7.23 (tt, *J* = 7.3, 2.2 Hz, 1H), 7.16 (dd, *J* = 5.1, 3.6 Hz, 1H).

2-phenyl-1H-phenanthro[9,10-d]imidazole (3i)

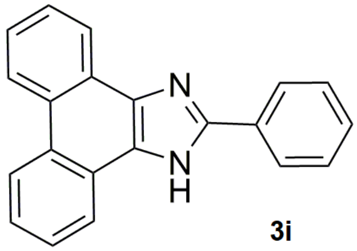

3i

Mp: >260 °C (311-313 °C)[4]; ^{1}H NMR (400 MHz, DMSO) δ 13.47 (s, 1H), 8.87 (dd, *J* = 15.1, 8.7 Hz, 1H), 8.64 – 8.64 (m, 2H), 8.32 (dd, *J* = 7.6, 0.6 Hz, 2H), 7.83 – 7.68 (m, 2H), 7.68 – 7.57 (m, 4H), 7.51 (t, *J* = 7.3 Hz, 1H).

2-(4-bromophenyl)-1H-phenanthro[9,10-d]imidazole (3j)

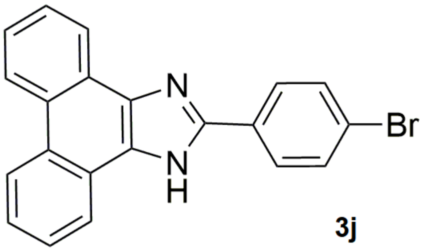

3j

Mp: >260 °C (280-282 °C)[4]; ^{1}H NMR (400 MHz, DMSO) δ 13.57 (s, 1H), 8.88 (d, *J* = 8.3 Hz, 2H), 8.56 (d, *J* = 7.9 Hz, 2H), 8.27 (d, *J* = 8.5 Hz, 2H), 7.83 (d, *J* = 8.5 Hz, 2H), 7.76 (t, *J* = 7.3 Hz 2H), 7.66 (t, *J* = 7.3 Hz, 2H).

2-(4-chlorophenyl)-1H-phenanthro[9,10-d]imidazole (3k)

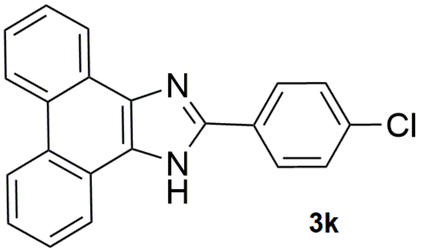

3k

Mp: >260 °C (274-276 °C)[4]; ^{1}H NMR (400 MHz, DMSO) δ 12.00 (s, 1H), 8.88 (dd, *J* = 14.0, 8.3 Hz, 2H), 8.56 (dd, *J* = 16.0, 7.8 Hz, 2H), 8.34 (d, *J* = 8.2 Hz, 2H), 7.82 – 7.58 (m, 6H).

2-(2-chloro-6-fluorophenyl)-1H-phenanthro[9,10-d]imidazole (3l)

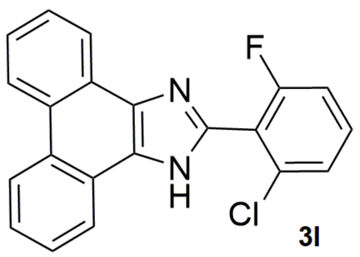

3l

Mp: >260 °C; ^{1}H NMR (400 MHz, DMSO) δ 13.80 (s, 1H), 8.90 (dd, *J* = 15.8, 8.3 Hz, 2H), 8.53 (d, *J* = 7.7 Hz, 1H), 8.36 (d, *J* = 7.8 Hz, 1H), 7.79 – 7.59 (m, 6H), 7.53 (t, *J* = 8.8 Hz, 1H).

2-(4-nitrophenyl)-1H-phenanthro[9,10-d]imidazole (3m)

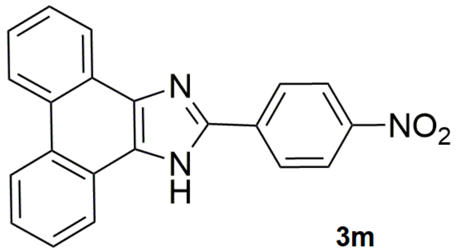

3m

Mp: >260 °C (335-337 °C)[4]; ^{1}H NMR (400 MHz, DMSO) δ 13.85 (s, 1H), 8.93 – 8.82 (m, 2H), 8.65 – 8.53 (m, 4H), 8.48 (d, *J* = 9.0 Hz, 2H), 7.85 – 7.62 (m, 4H).

2-(2-nitrophenyl)-1H-phenanthro[9,10-d]imidazole (3n)

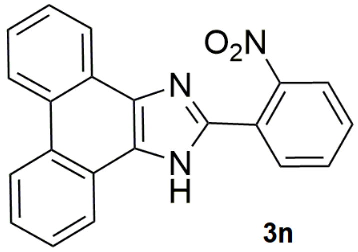

3n

Mp: >260 °C (267 °C)[5]; ^{1}H NMR (400 MHz, DMSO) δ 13.79 (s, 1H), 8.88 (d, *J* = 7.5 Hz, 2H), 8.44 (d, *J* = 7.7 Hz, 2H), 8.17 – 8.06 (m, 2H), 7.93 (t, *J* = 7.5 Hz, 1H), 7.83 – 7.61 (m, 5H).

2-(4-isopropylphenyl)-1H-phenanthro[9,10-d]imidazole (3o)

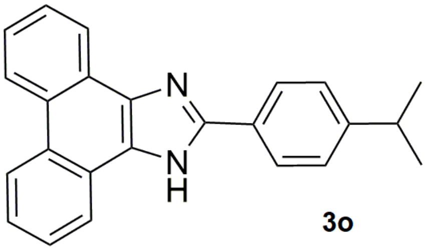

3o

Mp: >260 °C; ^{1}H NMR (400 MHz, DMSO) δ 13.38 (s, 1H), 8.87 (dd, *J* = 12.0, 7.3 Hz, 2H), 8.57 (t, *J* = 8.4 Hz, 2H), 8.24 (d, *J* = 7.9 Hz, 2H), 7.82 – 7.68 (m, 2H), 7.68 – 7.60 (m, 2H), 7.48 (d, *J* = 7.9 Hz, 2H), 3.01 (septet, *J* = 7.6, Hz, 2H), 1.29 (t, *J* = 8.3, Hz, 1H).

2-([1,1'-biphenyl]-4-yl)-1H-phenanthro[9,10-d]imidazole (3p)

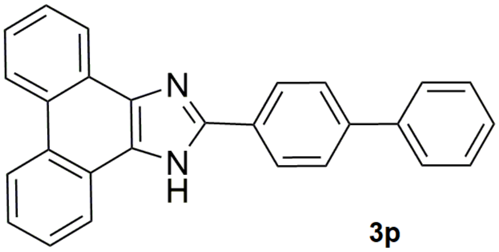

3p

Mp: >260 °C (258-259 °C)[6]; ^{1}H NMR (400 MHz, DMSO) δ 13.54 (s, 1H), 8.89 (dd, *J* = 13.3, 8.4 Hz, 2H), 8.61 (dd, *J* = 14.2, 7.9 Hz, 2H), 8.42 (d, *J* = 8.4 Hz, 2H), 7.95 (d, *J* = 8.4 Hz, 2H), 7.86 – 7.71 (m, 4H), 7.71 – 7.64 (m, 2H), 7.54 (t, *J* = 7.4, Hz, 2H), 7.43 (t, *J* = 7.4, Hz, 1H).

4-nitro-2-(1H-phenanthro[9,10-d]imidazol-2-yl)phenol (3q)

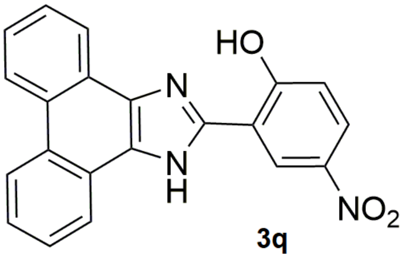

3q

Mp: >260 °C (259-260 °C)[7]; ^{1}H NMR (400 MHz, DMSO) δ 14.32 (s, 1H), 9.24 (d, *J* = 2.7 Hz, 1H), 8.89 (d, *J* = 8.2 Hz, 2H), 8.51 (dd, *J* = 7.9, 1.0 Hz, 2H), 8.23 (dd, *J* = 9.1, 2.7 Hz, 1H), 7.79 (dt, *J* = 7.4, 0.7 Hz, 2H), 7.70 (dt, *J* = 8.2, 1.3 Hz, 2H), 7.23 (d, *J* = 9.1 Hz, 1H).

2-(4-methoxyphenyl)-1H-phenanthro[9,10-d]imidazole (3r)

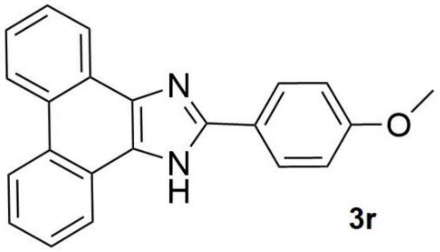

3r

Mp: 252-254 °C (254-255 °C)[8]; ^{1}H NMR (400 MHz, DMSO) δ 13.31 (s, 1H), 8.93 – 8.81 (m, 2H), 8.64 – 8.50 (m, 2H), 8.26 (dd, *J* = 6.9, 1.9 Hz, 2H), 7.84 – 7.68 (m, 2H), 7.64 (t, *J* = 7.2 Hz, 2H), 7.18 (d, *J* = 8.8 Hz, 2H), 3.88 (s, 3H).

2. ^{1}H NMR of compounds

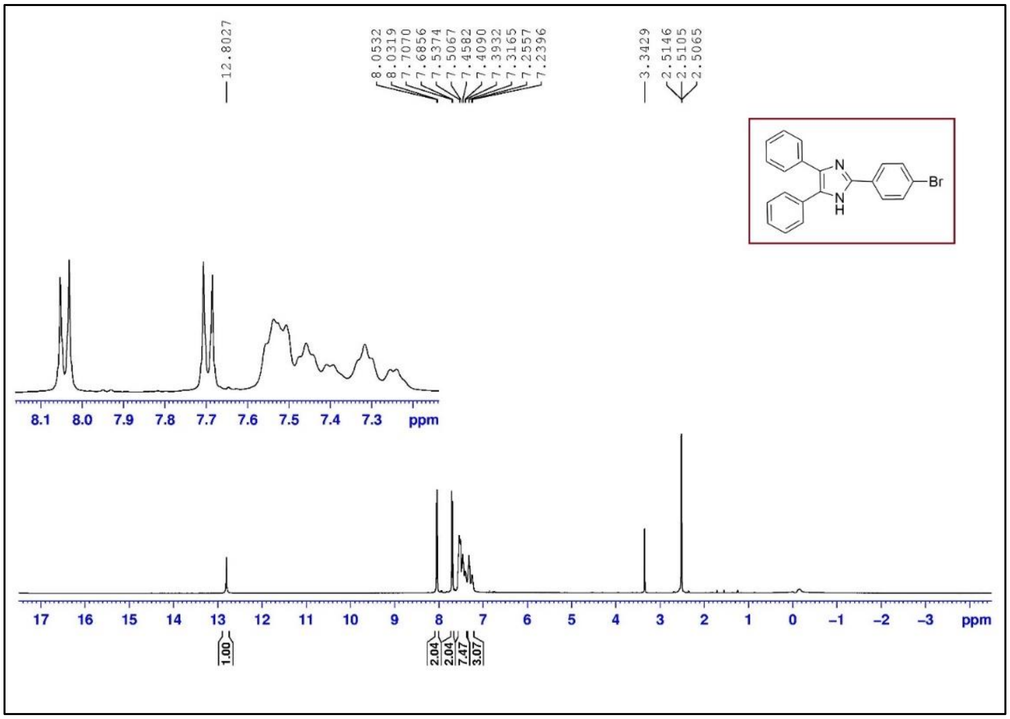

Figure S1. ^{1}H NMR spectrum of compound (3a) in DMSO.

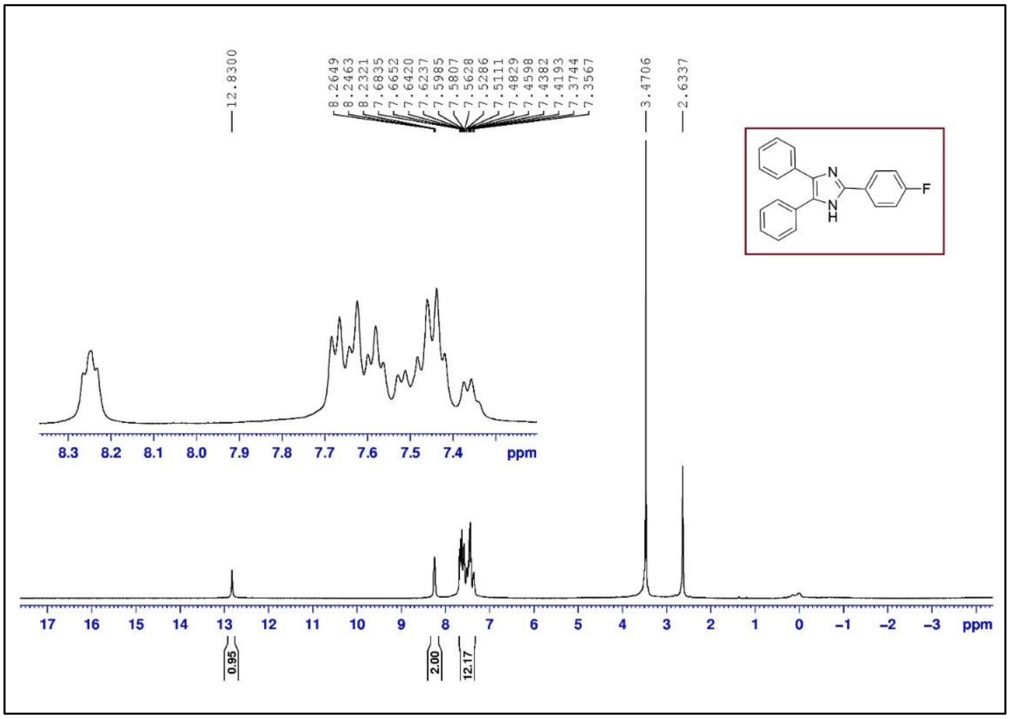

Figure S2. ^{1}H NMR spectrum of compound (3b) in DMSO.

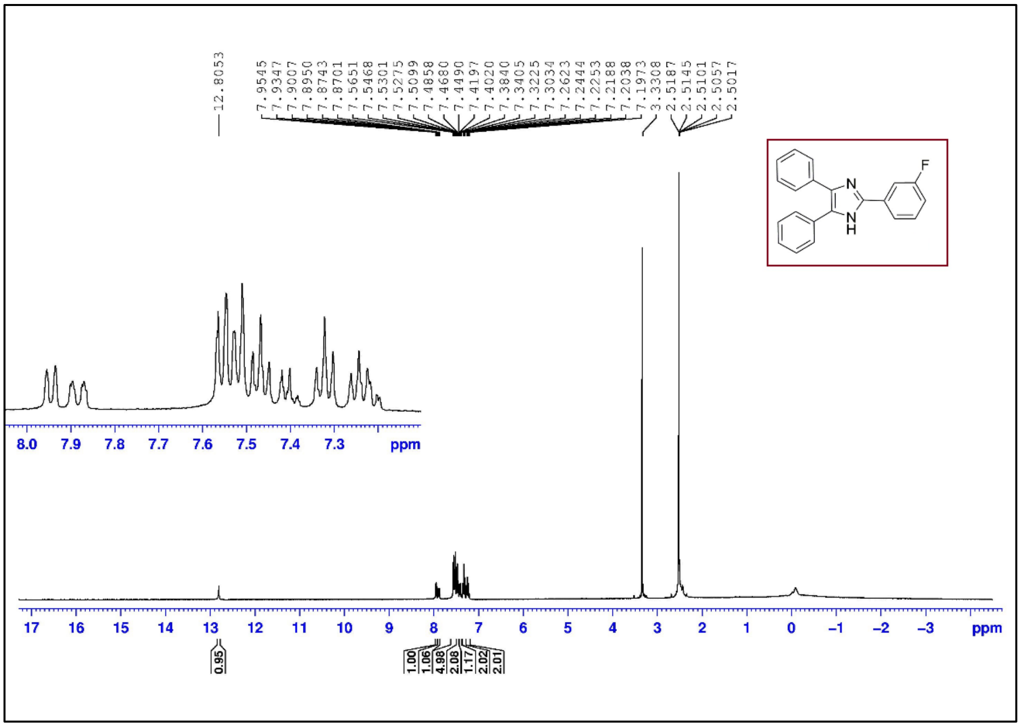

Figure S3. ^{1}H NMR spectrum of compound (3c) in DMSO.

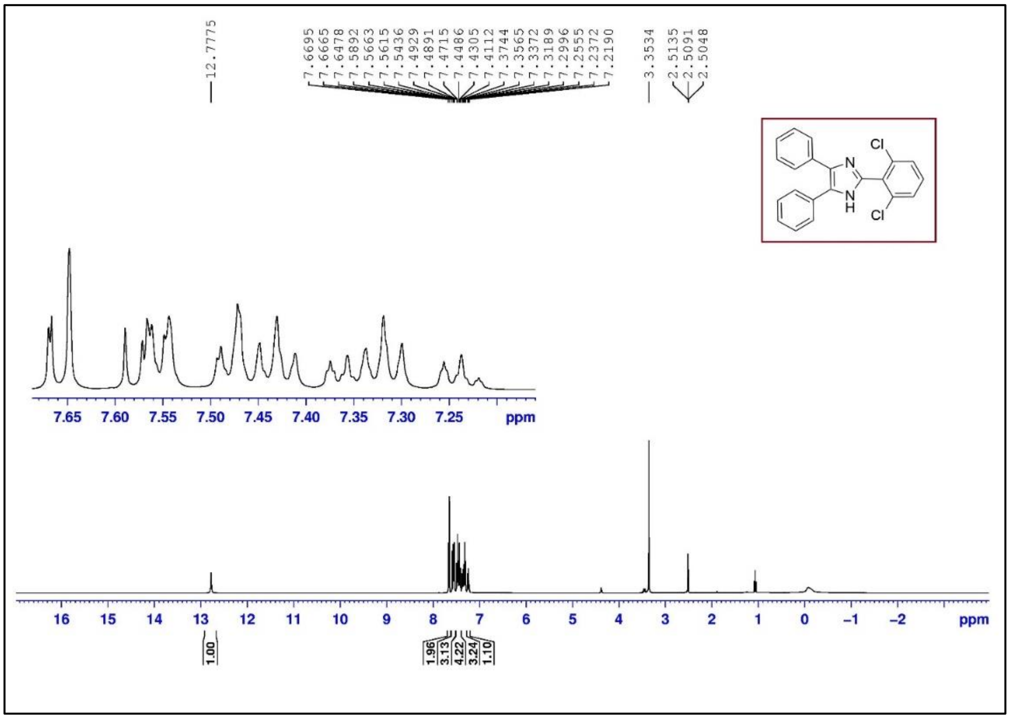

Figure S4. ^{1}H NMR spectrum of compound (3d) in DMSO.

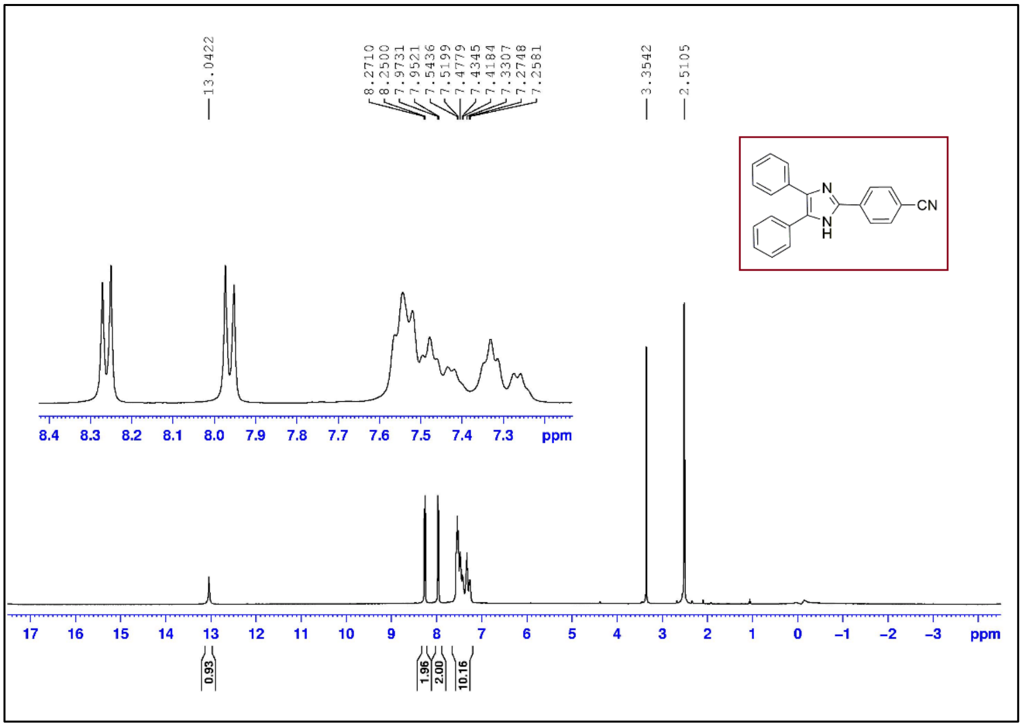

Figure S5. ¹H NMR spectrum of compound (3e) in DMSO.

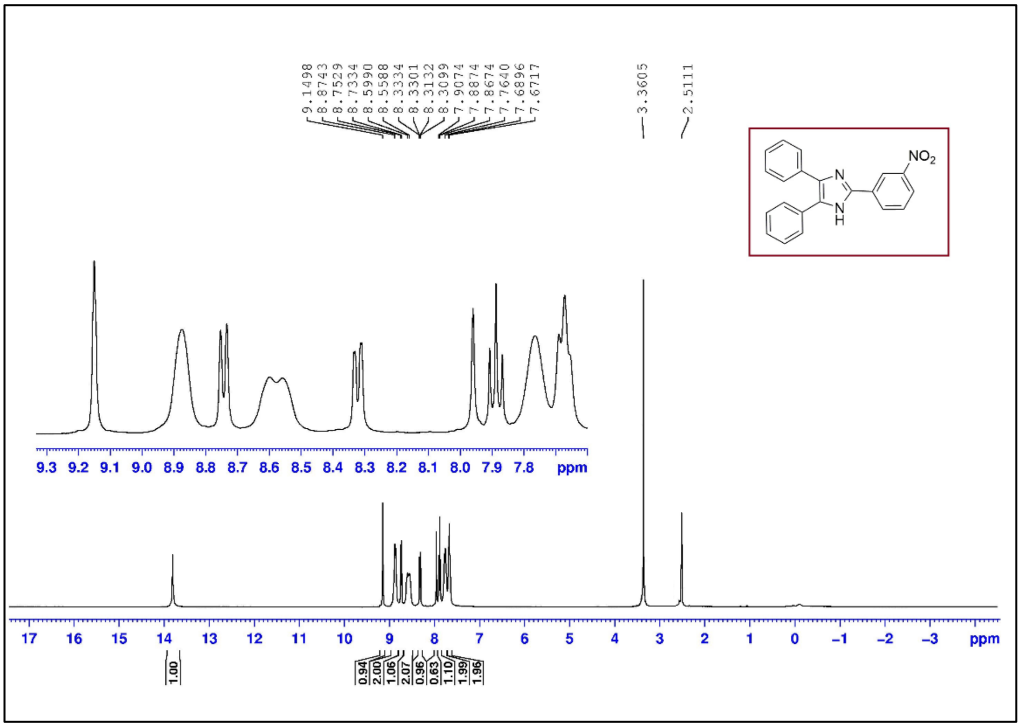

Figure S6. ¹H NMR spectrum of compound (3f) in DMSO.

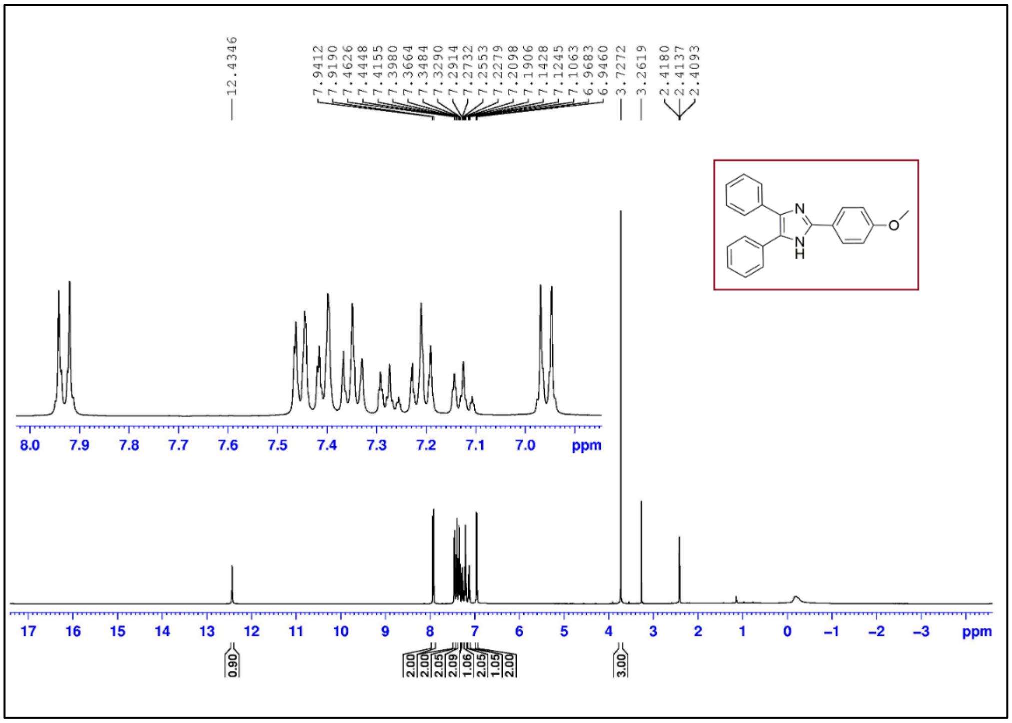

Figure S7. ^{1}H NMR spectrum of compound (4g) in DMSO.

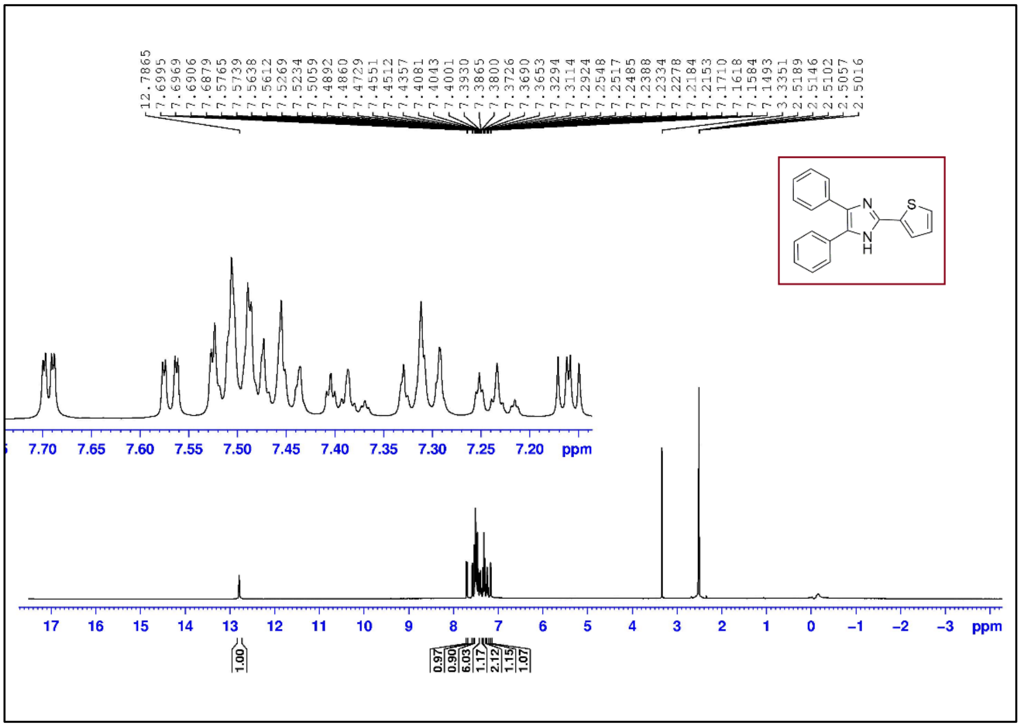

Figure S8. ^{1}H NMR spectrum of compound (3h) in DMSO.

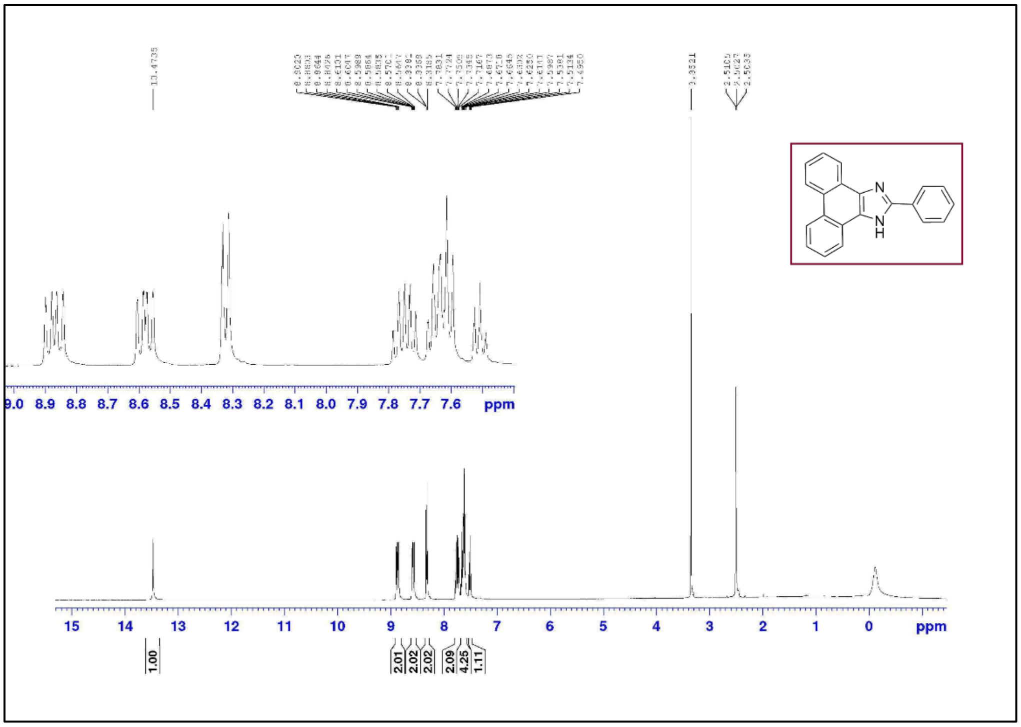

Figure S9. ^{1}H NMR spectrum of compound (3i) in DMSO.

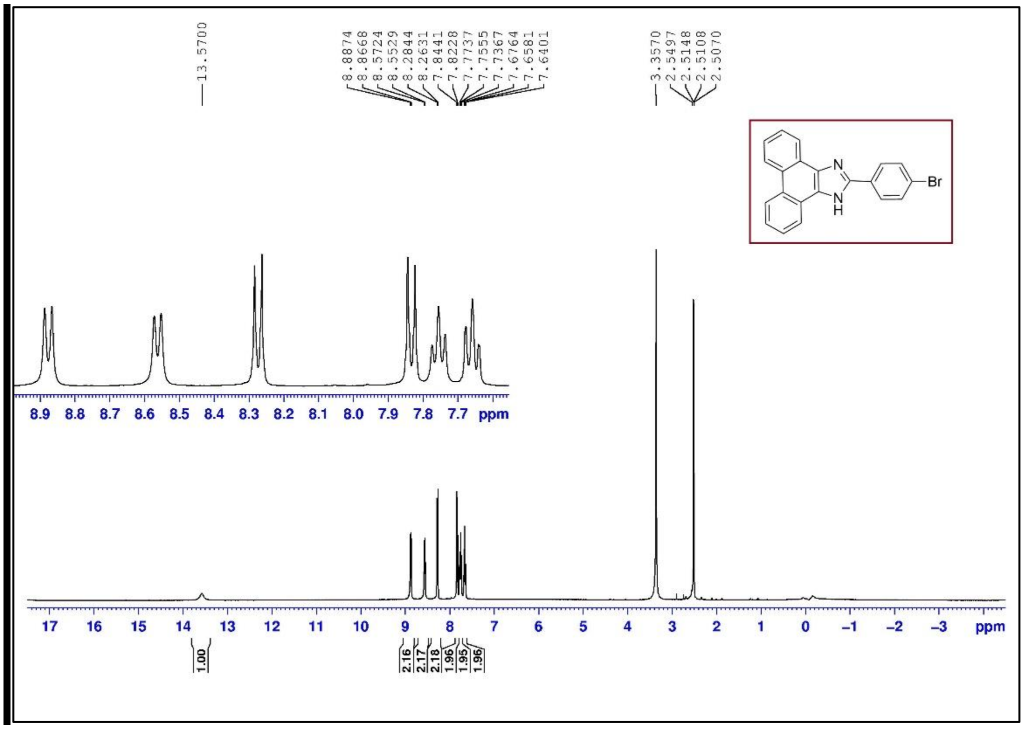

Figure S10. ^{1}H NMR spectrum of compound (3j) in DMSO.

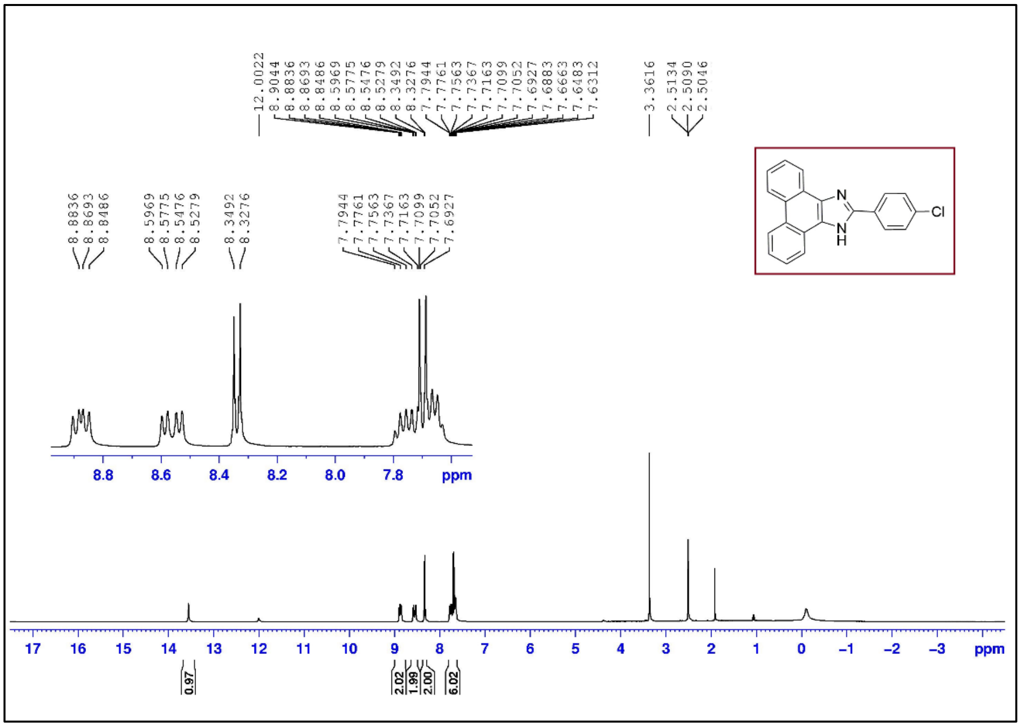

Figure S11. ^{1}H NMR spectrum of compound (3k) in DMSO.

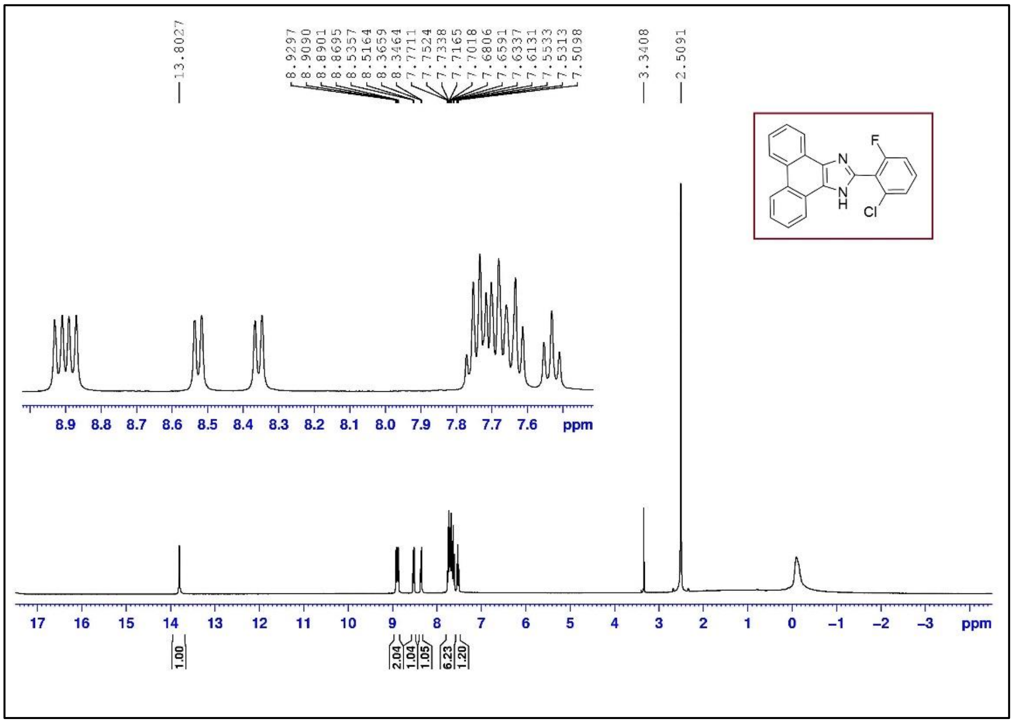

Figure S12. ^{1}H NMR spectrum of compound (3l) in DMSO.

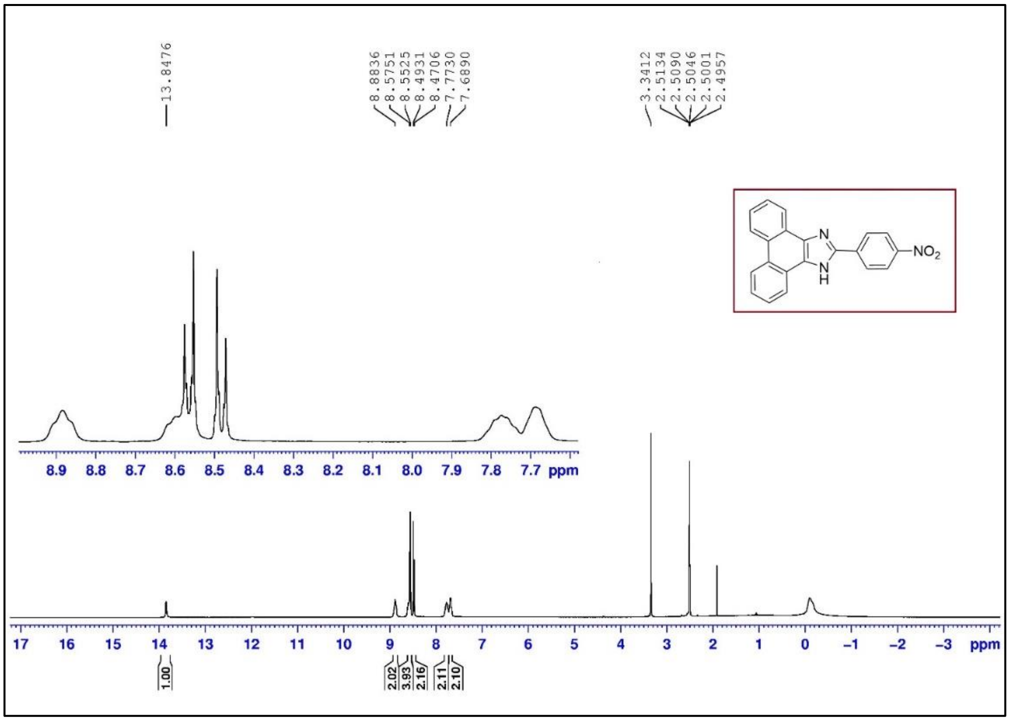

Figure S13. ^{1}H NMR spectrum of compound (3m) in DMSO.

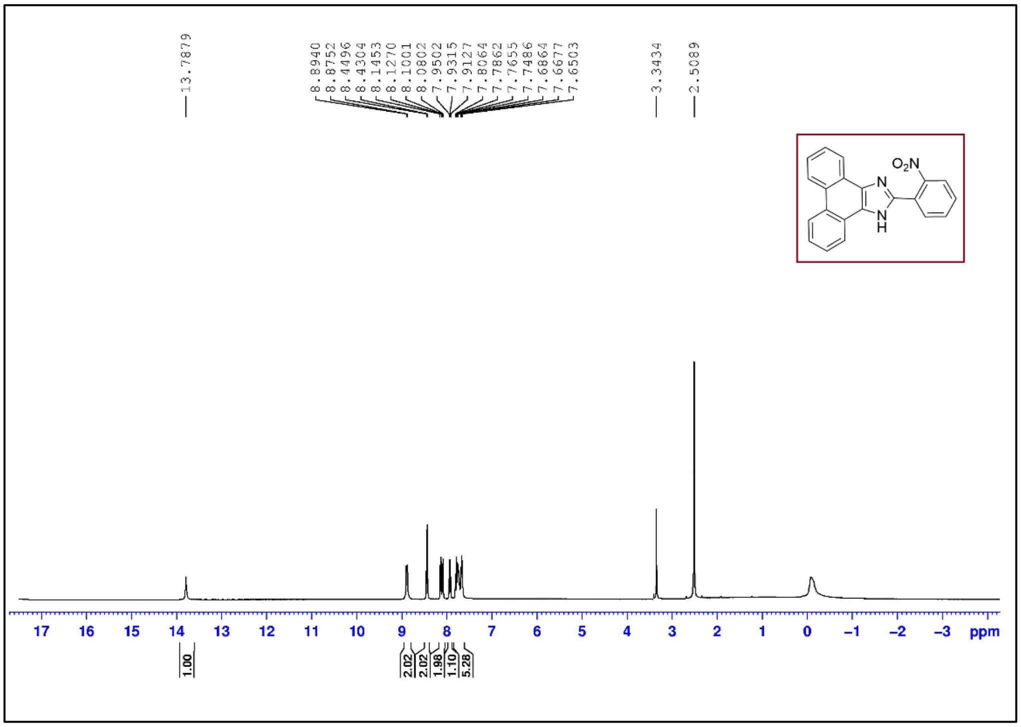

Figure S14. ^{1}H NMR spectrum of compound (3n) in DMSO.

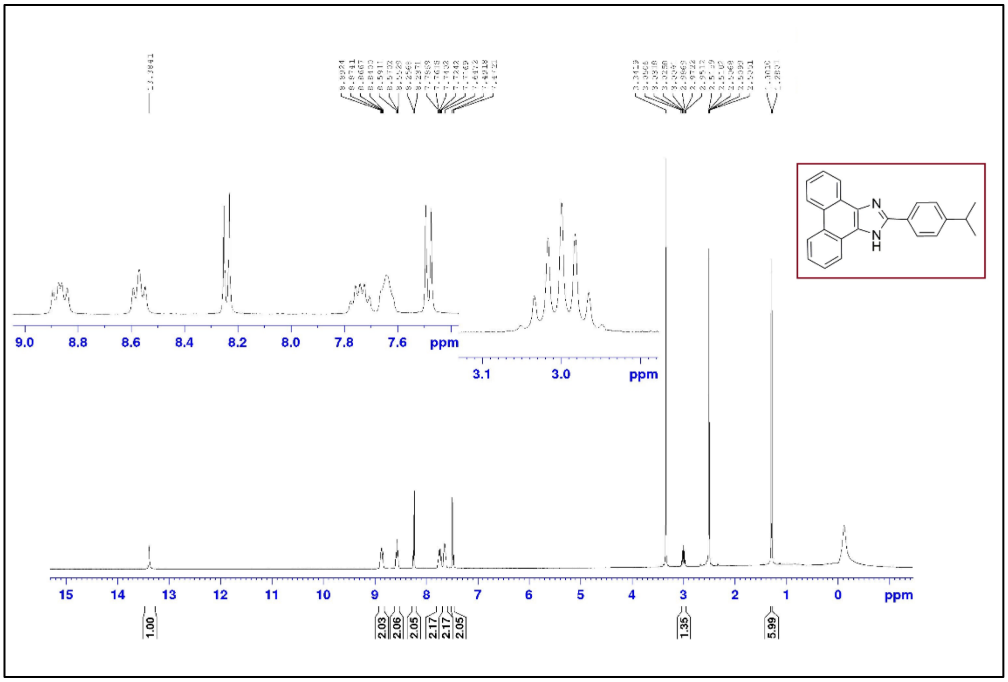

Figure S15. ^{1}H NMR spectrum of compound (3o) in DMSO.

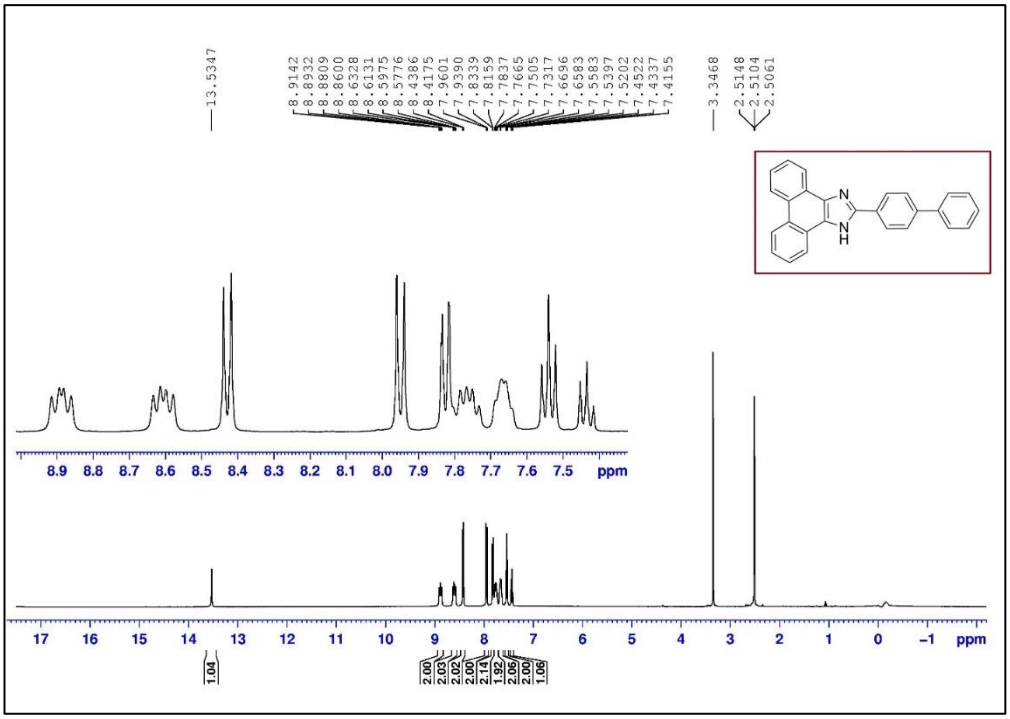

Figure S16. ^{1}H NMR spectrum of compound (3p) in DMSO.

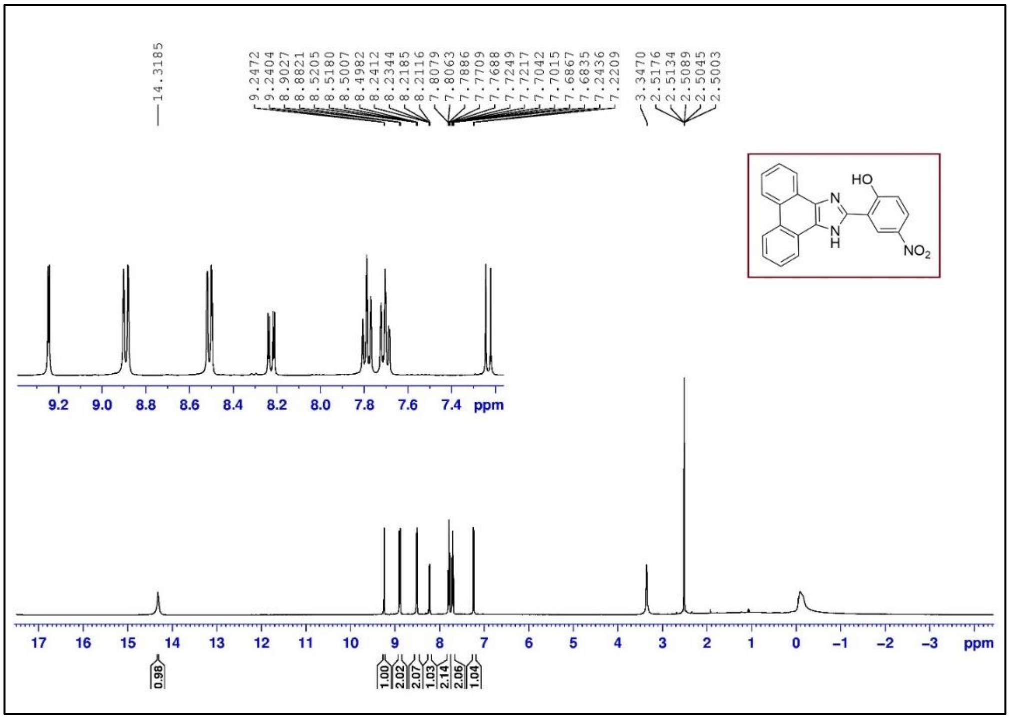

Figure S17. ^{1}H NMR spectrum of compound (3q) in DMSO.

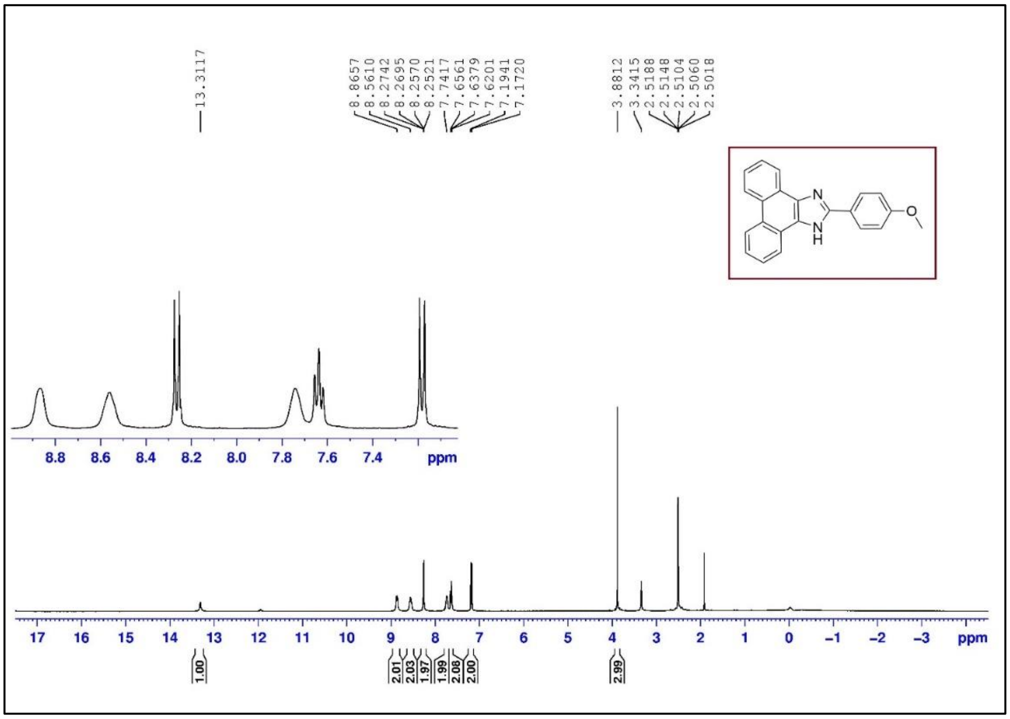

Figure S18. ^{1}H NMR spectrum of compound (3r) in DMSO.

References

[1] B. Das, J. Kashanna, R.A. Kumar, P. Jangili, Synthesis of 2, 4, 5-trisubstituted and 1,2,4,5-tetrasubstituted imidazoles in water using *p*-dodecylbenzenesulfonic acid as catalyst, Monatshefte Für Chemie-Chemical Mon. 144 (2013) 223–226. https://doi.org/10.1007/s00706-012-0770-0.

[2] J. Jayram, V. Jeena, Copper-catalyzed aerobic benzylic sp 3 C–H oxidation mediated synthesis of 2,4,5-trisubstituted imidazoles via a domino multi-component reaction, Green Chem. 19 (2017) 5841–5845. https://doi.org/10.1039/c7gc02484c.

[3] F. Xu, N. Wang, Y. Tian, G. Li, Simple and efficient method for the synthesis of highly substituted imidazoles catalyzed by benzotriazole, J. Heterocycl. Chem. 50 (2013) 668–675.

[4] N.L. Higuera, D. Peña-Solórzano, C. Ochoa-Puentes, Urea–zinc chloride eutectic mixture-mediated one-pot synthesis of imidazoles: efficient and ecofriendly access to trifenagrel, Synlett. 30 (2019) 225–229. https://doi.org/10.1055/s-0037-1610679.

[5] A.H. Cook, D.G. Jones, 49. Experiments in the triazine and the glyoxaline series, J. Chem. Soc. (1941) 278–282.

[6] O. Neunhoeffer, B. Krieg, Über die Chemolumineszenz Lophin-analoger Verbindungen, Zeitschrift Für Naturforsch. B. 21 (1966) 536–539. https://doi.org/10.1515/znb-1966-0608.

[7] A.O. Eseola, O. Akogun, H. Görls, O. Atolani, G.A. Kolawole, W. Plass, Ligand characteristics and in situ generation of Pd active species towards CC coupling using series of 2-(1*H*-imidazol-2-yl)phenols, J. Mol. Catal. A Chem. 387 (2014) 112–122. https://doi.org/10.1016/j.molcata.2014.02.032.

[8] H. Eshghi, M. Rahimizadeh, M. Hasanpour, M. Bakavoli, A novel imidazolium-based acidic ionic liquid as an efficient and reusable catalyst for the synthesis of 2-aryl-1*H*-phenanthro[9,10-d]imidazoles, Res. Chem. Intermed. 41 (2015) 4187–4197. https://doi.org/10.1007/s11164-013-1522-4.
